# Turn it off and on again: characteristics and control of torpor

**DOI:** 10.12688/wellcomeopenres.17379.1

**Published:** 2021-11-17

**Authors:** Michael Ambler, Timna Hitrec, Anthony Pickering

**Affiliations:** 1 School of Physiology, Pharmacology, & Neuroscience, University of Bristol, Bristol, Bristol, BS8 1TD, UK

**Keywords:** Torpor, Metabolism, Homeostasis, Thermoregulation, Hibernation, Energy balance

## Abstract

Torpor is a hypothermic, hypoactive, hypometabolic state entered into by a wide range of animals in response to environmental challenge. This review summarises the current understanding of torpor. We start by describing the characteristics of the wide-ranging physiological adaptations associated with torpor. Next follows a discussion of thermoregulation, control of food intake and energy expenditure, and the interactions of sleep and thermoregulation, with particular emphasis on how those processes pertain to torpor. We move on to take a critical view of the evidence for the systems that control torpor entry, including both the efferent circulating factors that signal the need for torpor, and the central processes that orchestrate it. Finally, we consider how the putative circuits responsible for torpor induction integrate with the established understanding of thermoregulation under non-torpid conditions and highlight important areas of uncertainty for future studies.

## Introduction

Torpor is a hypothermic, hypometabolic, adaptive response, observed when the metabolic demands of maintaining homeostasis cannot be met. Torpor can be brief, in daily heterotherms such as the mouse, or it can be prolonged, in seasonal hibernators. In small animals undergoing torpor, body temperature typically drops to just a few degrees above ambient. In the hibernating arctic squirrel this can result in core temperatures as low as -2.9°C ( Barnes, 1989). Alongside hypothermia are commensurate reductions in metabolic, heart, and respiratory rates ( Heldmaier *et al*., 2004). Torpor therefore represents an intriguing deviation from ‘normal’ mammalian physiology, and as such there is increasing interest in mimicking it in the context of the critically ill patient ( Cerri, 2017; Stanzani *et al*., 2020), and for long-distance space travel ( Cerri *et al*., 2021a).

**Figure 1.**
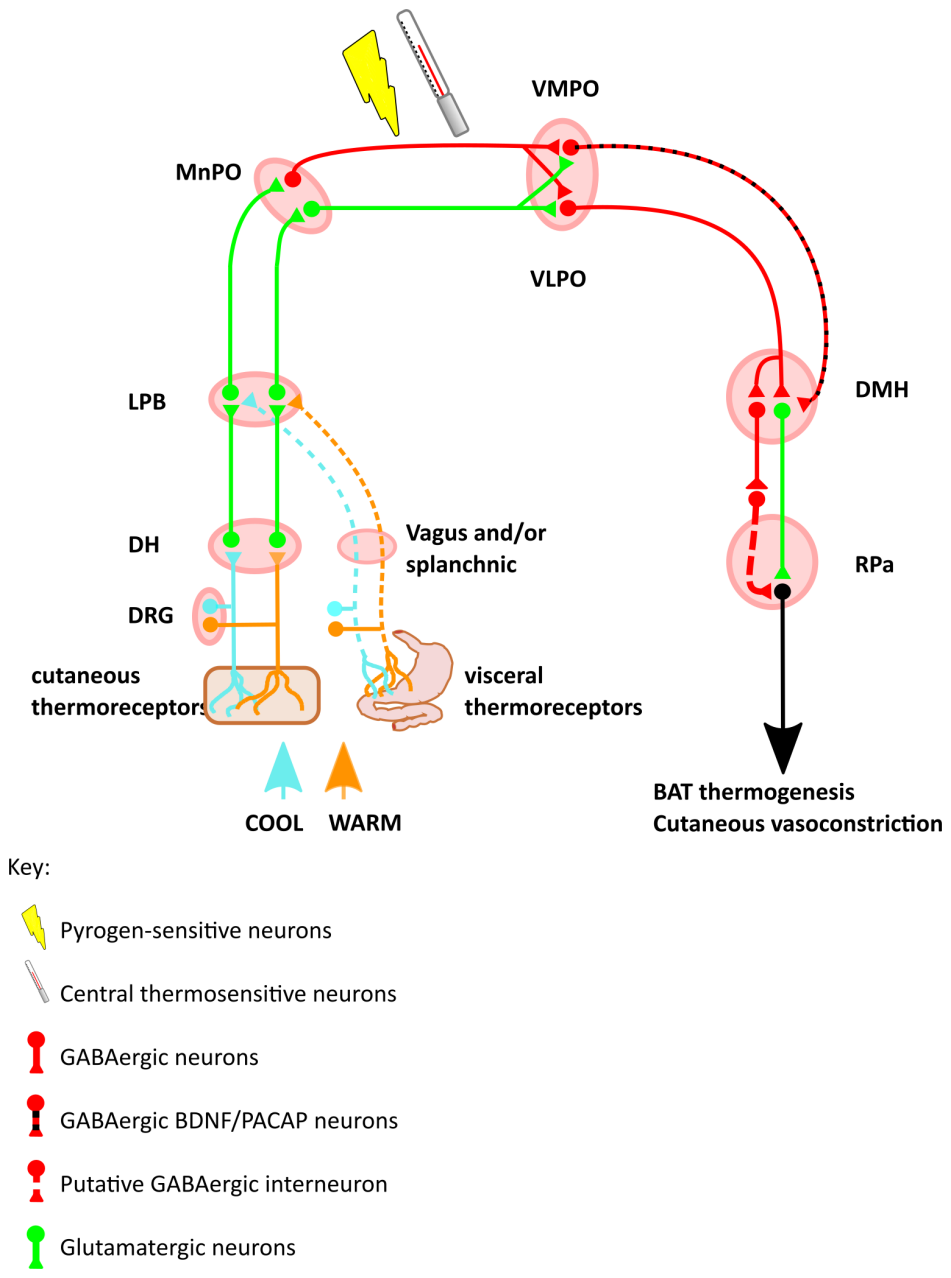
Schematic of the thermal defence circuit. Inputs include skin surface thermoreceptors, visceral thermoreceptors, intrinsic thermosensitive neurons in the POA, and circulating pyrogens. Warm-sensing neurons increase the inhibitory input from the VMPO and VLPO onto the DMH, which reduces a tonically active thermogenic signal from DMH to RPa. Cool-sensing neurons disinhibit this tonically active DMH to RPa projection, to increase heat production. Abbreviations: DRG, dorsal root ganglion; DH, dorsal horn; LPD, lateral parabrachial nucleus; MnPO, median preoptic area; VLPO, ventrolateral preoptic nucleus; VMPO, ventromedial preoptic nucleus; BDNF, brain-derived neurotrophic factor; PACAP, pituitary adenylate cyclase-activating polypeptide; DMH, dorsomedial hypothalamic nucleus; RPa, raphe pallidus; BAT, brown adipose tissue.

Torpor (or aestivation in the case of fish, reptiles, and amphibians) is a remarkably conserved behaviour seen in all classes of vertebrate life, including the three oldest branches of mammals: monotremes, marsupials, and placentals including primates, as illustrated in Figure 1 ( Carey *et al*., 2003; Heldmaier *et al*., 2004). Those mammals that engage torpor, do so through activation of ubiquitous genes, rather than a complement of genes that are unique to those torpid species ( Faherty *et al*., 2016; Srere *et al*., 1992; Zhao *et al*., 2010). Hence, this behaviour may represent a fundamental physiological response that has been relatively recently switched off in those animals for which it is not an extant behaviour. The aim of this review is to provide an overview of the fundamental physiological adaptations associated with torpor, and then to discuss current evidence for how these remarkable adjustments might be achieved. We focus on the physiology of daily torpor in the mouse, primarily because this is the animal that has been most extensively studied. However, where relevant or where data in the mouse are lacking, other species including hamsters, squirrels, bears, and lemurs will be included. The latter group undergo prolonged seasonal hibernation, and an assumption is that the torpor during these hibernation periods is mechanistically equivalent to the brief torpor bouts seen in daily heterotherms such as the mouse. However, this assumption has not been experimentally proven.

## Discussion

### Physiological characteristics of torpor

Due to the large surface area to volume ratio of a mouse, maintenance of normothermia represents a significant energy cost. In mice housed at an ambient temperature of 22°C, over 30% of energy expenditure is directed towards thermogenesis ( Abreu-Vieira *et al*., 2015). When mice are unable to access sufficient calories to maintain active physiology and normal body temperature, they will enter periods of torpor lasting several hours ( Hudson & Scott, 1979). Stimuli for torpor in mice include acute fasting ( Sunagawa & Takahashi, 2016), a combination of fasting and reduction in the ambient temperature ( Hitrec *et al*., 2019; Swoap & Gutilla, 2009), food restriction ( van der Vinne *et al*., 2018), or simply increasing the energy costs of foraging ( Schubert *et al*., 2010). This latter observation supports the hypothesis that it is a relative imbalance of energy supply compared to the demands of maintaining ‘normal’ physiological homeostasis rather than a response to cold and hunger per se, which triggers torpor. In summary: torpor is engaged in any situation where the available food is insufficient for the metabolic demands of euthermia.

Female mice are more prone to torpor than males, and hence some studies present data from female mice only (for example, Kato *et al*., 2018; Oelkrug *et al*., 2010; Swoap & Gutilla, 2009) although males do enter torpor ( Gavrilova *et al*., 1999; Lo Martire *et al*., 2018; Solymár *et al*., 2015; Sunagawa & Takahashi, 2016). Recent work indicates oestrogen receptor expressing neurons in the pre-optic area contribute to the generation of daily torpor in the mouse, and will be discussed in more detail later ( Zhang *et al*., 2020). Evidence from the pouched mouse, *Saccostomus campestris,* indicates that testosterone inhibits torpor ( Mzilikazi & Lovegrove, 2002). These gender differences might reflect the relatively lower energetic burden that normal activity and reproduction place on male compared to female mice, hence the need to conserve adipose tissue energy reserves may be greater for females. Alternatively, the relative female predisposition to torpor may be due to lower birth weight, since low birth weight predicts higher torpor tendency irrespective of actual body weight at the time of fasting ( Kato *et al*., 2018).

### Definition, timing, and duration of torpor

The transition to torpor may not be all or nothing: fasted mice exhibit increased variability of both metabolic rate and body temperature, with graded reductions in core body temperature up to full torpor ( Brown & Staples, 2010). Despite the magnitude of the final deviation from normal physiology, there is no consensus on the definition of torpor in mice. Examples include a core body temperature below 34°C proceeded by least fifteen minutes of consecutive decline ( Iliff & Swoap, 2012; Willis, 2007); body temperature below 31°C for at least 30 minutes ( Brown & Staples, 2010); body temperature below 32°C ( Braulke & Heldmaier, 2010); a metabolic rate 25% below expected ( Hudson & Scott, 1979); the duration of a period of monotonic cooling resulting in a reduction in body temperature of at least 5°C followed by a period of monotonic increase up to at least 5°C above the nadir ( Lo Martire *et al*., 2018); or, deviation from a Bayesian estimate of individual basal metabolic rate or core temperature ( Sunagawa & Takahashi, 2016). These approaches vary in their complexity, as well as their ability to account for individual and/or circadian fluctuations in body temperature or metabolic rate.

Although the trigger for torpor entry in mice usually originates from a fluctuating rather than predictable environmental stimulus, the timing of torpor entry is under circadian control. Torpor in mice generally occurs during the latter part of the lights off period ( Brown & Staples, 2010; Webb *et al*., 1982). Timing of torpor entry is primarily under the control the circadian clock, but can be adjusted by the timing or expected timing of food ( van der Vinne *et al*., 2018), or entrained to food in mice or hamsters lacking endogenous circadian clocks ( Paul *et al*., 2004; van der Vinne *et al*., 2018). Duration of torpor in mice is inversely proportional to the weight of the mouse ( Hudson & Scott, 1979), with some evidence that torpor is engaged when food restriction or fasting decreases body weight to approximately 20g ( Solymár *et al*., 2015), although this experiment was performed only in male mice. Torpor typically persists for between four and six hours, often preceded by shallow, aborted or shorter bouts ( Hudson & Scott, 1979; Webb *et al*., 1982). Bouts may last 12 hours or more depending on the intervention used to induce torpor ( Kato *et al*., 2018).

### Thermoregulation during torpor

During torpor bouts, thermoregulation is not simply suspended: mice maintain active control of their temperature, usually tracking approximately two degrees above ambient temperature, but defending a minimum body temperature of 16 - 19°C ( Hudson & Scott, 1979). Further evidence for the continued - albeit adjusted - thermoregulatory control comes from the observation that the rate of decline in body temperature is lower in torpor than when hypothermia is induced pharmacologically or physically ( Vicent *et al*., 2017b), that is to say, temperature decreases during entry into torpor are controlled. Indeed, a very low ambient temperature may reduce the probability of torpor entry, again indicating ongoing but adjusted thermoregulation ( Sunagawa & Takahashi, 2016). Hypothermia in a torpid animal could be achieved through three distinct mechanisms: increased thermal conductance to the environment; reduced target temperature; or reduced gain in the regulatory feedback system. Hibernating marmots appear to reduce both the gain and the target of the thermoregulatory system ( Florant & Heller, 1977), while daily torpor in mice may predominantly involve reduction in its gain ( Sunagawa & Takahashi, 2016). Whether these observations reflect mechanistic differences between the hypothermia seen during daily torpor and that during torpor in hibernation is not clear. 

### Cardiovascular function during torpor

The suppression of metabolism associated with torpor allows animals to tolerate profound reductions in cardiac output, respiratory rate, and, presumably, organ perfusion. Heart rates of torpid mice typically reach a nadir of approximately 150 beats per minute (bpm) from resting rates of around 600 bpm ( Swoap & Gutilla, 2009). Hypothermia alone generates a degree of bradycardia, such as is seen clinically during therapeutic hypothermia ( Stær-Jensen *et al*., 2014). However, heart rates are slower at any given core body temperature during entry into, compared to arousal from torpor ( Swoap & Gutilla, 2009). This indicates a dominance of the parasympathetic nervous system during entry, followed by sympathetic nervous system activation during arousal from torpor, at least in terms of the heart. For a given body temperature, pharmacologically-induced hypothermia generates a less profound bradycardia than that seen in torpor, which again supports the hypothesis that heart rate is actively suppressed during torpor entry ( Vicent *et al*., 2017b).

Given that heart rate in torpid mice drops by 75% from resting values, and mean arterial blood pressure is determined by the product of cardiac output vascular resistance, if all other parameters remained the same a 75% drop in mean arterial pressure would be predicted during torpor. However, systolic, diastolic, and mean arterial pressure drop by only 25–30% during torpor ( Swoap & Gutilla, 2009). The maintenance of blood pressure at values only 25-30% below those seen in euthermic mice despite the profound bradycardia suggests significantly increased vascular resistance.

Hence, during torpor there appears to be simultaneous activation of the sympathetic and parasympathetic nervous system with the former driving vasoconstriction and the latter driving bradycardia. Simultaneous activation of both the sympathetic and parasympathetic limbs of the autonomic nervous system has been proposed as a means to optimise cardiac function when pumping blood into a constricted vascular tree ( Paton *et al*., 2005), and is observed for example in the diving reflex ( Panneton, 2013).

### Metabolic rate and respiratory function during torpor

Respiratory function in torpid mice is less well studied, however, in the little pocket mouse *Perognathus longimembris* (a 7–11 gram rodent from the family Heteromyidae) daily torpor is associated with a reduction in respiratory minute volume to less than 2% of basal levels ( Withers, 1977). Dormice ( *Glis glis*) exhibit similar characteristics during torpor ( Elvert & Heldmaier, 2005). Both species increase the rate and decrease the depth of ventilation during entry and exit from torpor, a pattern that resembles panting. The purpose of this panting is unclear, but it may serve to increase heat loss during torpor entry, reduce the partial pressure of carbon dioxide in the blood prior to torpor, or expel accumulated carbon dioxide following torpor.

The assessment of acid-base balance and partial pressures of O _2_ and CO _2_ in hypothermic hibernating animals is complex: the *p*H of pure water is dependent on temperature, as are the dissociation constants of biological buffers, and the partial pressure of a fixed amount of carbon dioxide ( *P*CO _2_) in a solution decreases with decreasing temperature. One approach for assessing acid-base balance in hypothermic or hibernating animals is to take a sample of blood held in a sealed container, normalise the temperature and then measure the *p*H. This allows comparison with ‘normal’ values taken at 37° C. Blood taken from hibernating hamsters ( *Cricetus cricetus*) and warmed to 37°C in a sealed syringe, tends to have a *p*H between 6.9 and 7.15 compared to euthermic *p*H of approximately 7.36, and an arterial *P*CO _2_ between 17 and 24 kilopascals (kPa) compared to euthermic values of approximately 6 kPa, indicating a respiratory acidosis. Alternatively, one can measure the dissociation ratio of imidazole groups on proteins (α _im_): acid conditions reduce the dissociation ratio (as does reduced temperature). Using this measure also indicates an acidic intracellular state during hibernation in blood, brain and muscle of hibernating hamsters ( Malan *et al*., 1985; Malan, 1988). In contrast to the observation of a respiratory acidosis in hibernating hamsters, hibernating arctic ground squirrels appear to have reduced *P*CO _2_ compared to euthermic ground squirrels and rats ( Ma *et al*., 2005). This difference may reflect differences in the body temperature of these hibernating species, with greater metabolic suppression in the colder artic ground squirrel leading to less CO _2 _production, differences in the physiology of the species, or differences in the method for analysing the blood gases taken from hypothermic animals. The partial pressure of oxygen ( *P*O _2_) in hibernating arctic ground squirrels is actually higher than in euthermic animals, reflecting presumably the reduced oxygen consumption, and only dips below normal euthermic values during arousal when metabolic activity is at a peak ( Ma *et al*., 2005).

Oxygen consumption decreases to between 0.04 and 0.05ml O2.g ^-1^.hr ^-1^ in torpid pocket mice and dormice, which in the former is less than 1% of levels when housed at an ambient temperature of 10°C ( Elvert & Heldmaier, 2005; Withers, 1977). Larger mammals such as the Alaskan black bear, whose basal metabolic rate is generally lower than that observed in smaller animals, suppress metabolism to 25% of resting levels. Notably, the minimum oxygen consumption seen in hibernating bears is very similar to that seen in smaller mammals, reaching a nadir of 0.06 ml O2.g ^-1^.hr ^-1^ ( Toien *et al*., 2011). The fact that large seasonal hibernators achieve similar metabolic suppression despite significantly higher body temperature during hibernation compared to small daily heterotherms, indicates that the reduction in metabolic rate is not simply a passive consequence of lowered body temperature, but rather that metabolism is actively suppressed. This hypothesis is further supported by several observations: reductions in heart rate, respiratory rate and oxygen consumption precede decreases in core temperature in all animals studied, and is largely independent of ambient temperature ( Elvert & Heldmaier, 2005; Toien *et al*., 2011; Withers, 1977). Metabolic rate in torpid dunnarts is several times lower than that seen in a similar sized rat pup rendered hypothermic by exposure to a cold ambient temperature, with reversal of the normal relationship between body warming or cooling and metabolic rate during torpor ( Geiser *et al*., 2014). Respiration in mitochondria taken from torpid mice is suppressed even when assessed at 37°C ( Brown & Staples, 2010).

While metabolism is clearly suppressed during torpor, there is evidence that it increases immediately prior to torpor entry in the mouse ( Lo Martire *et al*., 2018), the dormouse ( Elvert & Heldmaier, 2005), and the Djungarian hamster ( Heldmaier *et al*., 1999). The significance of this is not clear, but it may represent incomplete switching from euthermia to torpor with resultant episodes of shivering, or perhaps there is a need to clear metabolic substrates from the mitochondria prior to torpor in order to suppress metabolism and reduce free radical production during torpor.

Remarkably, despite the dramatically reduced cardiac output and respiratory rate, hence presumably reduced oxygen delivery, the concentration of blood lactate in hibernating arctic ground squirrels is no different from that seen in euthermic controls ( Ma *et al*., 2005). This demonstrates the remarkable fine-tuning of metabolic supply and demand during torpor such that in the face of reduced supply there is no overall deficit.

### Physiology of mammalian thermoregulation

In order to understand the physiological adaptations of torpor, it is useful to understand the normal control of body temperature. Mammalian thermoregulation presumably evolved to enable control of the core temperature such that cellular and tissue function is optimised to the survival and reproductive benefit of the animal. The considerable energy cost of maintaining a core temperature several degrees above ambient implies homeothermy brings a significant survival advantage, allowing continued activity in the face of diurnal or seasonal reductions in the ambient temperature, and therefore allowing the occupation of a wider range of environmental niches ( Abreu-Vieira *et al*., 2015).

Despite this investment and its benefits, there are times when mammalian body temperature deviates from the normal range. This deviation can be physiological, such as during fever, sleep, ovulation, stress, or of course, torpor ( Landolt *et al*., 1995). Additionally, deviations of core temperature can be pathological due to poisoning or environmental challenges. Internal sources of heat in mammals include those that generate heat as a by-product, for example basal metabolism with heat generated as a consequence of pumping ions across membranes, and heat generated by muscles during movement. Additional internal sources of heat include those for which heat production is the primary goal. Examples of this include shivering, and brown adipose thermogenesis where mitochondrial proton flux is uncoupled from adenosine triphosphate (ATP) production in brown adipose tissue (reviewed in ( Nicholls & Rial, 1999)). In addition, mammals can take steps to reduce heat loss including piloerection, peripheral vasoconstriction, and nest building. Heat defence responses include cutaneous vasodilation with visceral constriction and increased cardiac output to direct blood towards the cooler skin surface, evaporative losses through sweating, panting, and grooming. Despite their mutually antagonistic effects, both the autonomic warm and cold defence responses are under the control of the sympathetic nervous system (reviewed in (( Morrison, 2016b)).

Early models of thermoregulation proposed a temperature target, or set-point, against which incoming signals of core and external temperature were compared, with a homeostatic response generated to drive the core temperature back towards the set-point ( Hammel & Pierce, 1968). More recently, with advances in our understanding of the physiology of thermoregulation, a model of multiple independent mechanisms each with their own threshold has emerged ( McAllen *et al*., 2010).

### Afferent thermoregulation signals

Signals encoding skin temperature arise from primary somatosensory neurons that express transient receptor potential (TRP) channels: the transducing receptor that converts temperature into electrochemical signals for neuronal transmission. Once transduced, the signal is passed to second order somatosensory neurons in the spinal dorsal horn ( Lumpkin & Caterina, 2007). Hot and cold information from the skin flows centrally in parallel streams, both relaying information from the dorsal horn via the parabrachial nucleus to the preoptic area of the hypothalamus (POA). Relatively little is known about the inputs from thermosensitive receptors in the viscera, although it is thought they enter the central nervous system (CNS) via splanchnic and vagal afferents, and follow a similar path through the lateral parabrachial nucleus to the preoptic area ( Morrison, 2016b)

The circuit controlling thermogenic responses to changes in skin temperature was identified by combining the use of retrograde tracer (cholera toxin B-subunit (CTb)), anterograde tracer ( *Phaseolus vulgaris* leucoagglutinin (PHA-L)), and immunohistochemistry against the c-Fos protein ( Nakamura & Morrison, 2008), a surrogate for neuronal activation ( Sagar *et al*., 1988). This anatomical analysis, as well as *in-vivo* electrophysiological recordings, indicate that the projection that travels via the external lateral and central parts of the lateral parabrachial nucleus (LPBel, LPBc, respectively) responds to skin cooling. Activation of this input to the preoptic area results in increased brown adipose tissue (BAT) thermogenesis, shivering, and increased metabolic and heart rate. The response is blocked by blockade of lateral parabrachial glutamate transmission, and does not require an intact spinothalamic tract. In contrast, skin warming induces c-Fos expression and increases firing in POA-projecting neurons in the dorsal part of the lateral parabrachial nucleus (LPBd). Activation of this pathway results in increased heart rate, suppressed BAT sympathetic nerve activity, and cutaneous vasodilatation, effects that are also dependent on glutamatergic transmission in the LPBd ( Nakamura & Morrison, 2010). Thermal-sensitive parabrachial neurons predominantly project to the median preoptic nucleus (MnPO) ( Nakamura & Morrison, 2008).

### Efferent thermoregulation signals

The POA can be viewed as sitting at the top of a thermoregulatory arc. It integrates information about both the internal and external temperature, and contributes to the autonomic response to thermal challenge by modulating BAT thermogenesis, shivering, and vasoconstriction ( Morrison *et al*., 2012; Morrison, 2016a). Early experiments established that local heating of the POA induced vasodilatation, sweating, and panting responses akin to those seen when heating the entire animal, indicating the existence of intrinsically warm-sensing POA neurons in central thermoregulatory circuits ( Clark *et al*., 1939b; Magoun *et al*., 1938; Nakayama *et al*., 1961). Lesioning the POA disrupts thermoregulatory responses to thermal challenge ( Clark *et al*., 1939a). In the 1970s, electrophysiological recordings confirmed that the POA not only responds to local brain temperature changes, but also responds to increases or decreases in skin surface or spinal temperature ( Boulant & Hardy, 1974). More recently, the application of agonists or antagonists to cultured POA neurons that express calcium-sensitive fluorescent dyes established that central temperature-sensing mechanisms are mediated by the transient receptor potential M2 channel (TRPM2). This channel, when exposed to increasing temperatures within the physiological range, allows influx of extracellular calcium ( Song *et al*., 2016). In summary, the POA receives thermal information from the skin and viscera, as well as directly sensing the local brain temperature, and then uses this information to control down-stream thermoregulation as discussed below.

Chemo- or optogenetic excitation of a warm-activated GABAergic projection from the ventral part of the lateral POA (vLPO) to the dorsomedial hypothalamic nucleus (DMH) suppresses thermogenesis and locomotion, while inhibiting the same projection induces the opposite effects. Data from *in-vivo* calcium imaging reveals the targets of this GABAergic projection are both glutamatergic and GABAergic neurons within the DMH, both of which increase their activity in response to low ambient temperature. Chemo- or optogenetic activation of either the GABAergic or the glutamatergic DMH neurons increases core temperature and activity ( Zhao *et al*., 2017). There is presumably a second inhibitory neuron between the DMH GABAergic neuron identified by Zhao *et al*., and the BAT-activating sympathetic premotor neurons in the raphe pallidus (RPa). This could in principle lie within either the DMH or the RPa or in a relay elsewhere (see Figure 1).

Another GABAergic projection to the DMH arises from the ventromedial preoptic area (VMPO). These VMPO neurons express brain-derived neurotrophic fact (BDNF) and pituitary adenylate cyclase-activating polypeptide (PACAP/PACAP). Again, *in-vivo* calcium imaging reveals that these VMPO neurons are activated by exposure to a warm environment. Opto-activation of the warm-sensing GABAergic BDNF and PACAP-expressing cell bodies in the VMPO induces a drop in core body temperature, vasodilatation, and preference for a cooler environment. Optoactivation in the DMH of the GABAergic terminals of these VMPO neurons results in a drop in core body temperature but no vasodilation or cool ambient preference. This implies that the DMH is responsible for the inhibition of BAT thermogenesis, whereas VMPO projections elsewhere generate the vasodilation and behavioural preference ( Tan *et al*., 2016).

The DMH then sends projections to the rostral raphe nucleus of the medulla that in turn project to, and activate, BAT via the spinal intermediolateral nucleus ( Cao *et al*., 2004; DiMicco & Zaretsky, 2007). This DMH-Raphe-BAT projection is involved in thermal defence and also in the thermogenic response to stress ( Kataoka *et al*., 2014). This implies that either the DMH receives inputs from additional regions beyond the POA thermo-sensitive circuit that mediate the stress response, or else that the POA also responds to stress.

Taken together, this work establishes the principle that the POA sends a GABAergic projection to the DMH to inhibit thermogenesis. With increasing ambient, hypothalamic, or core temperature, these signals increase to inhibit thermogenesis. Likewise, when internal and/or ambient temperature drops, activity in these GABAergic POA to DMH projections reduces, disinhibiting the DMH and leading to increased BAT thermogenesis. An additional principle that emerges is that the physiological effects of changes in temperature may be sensed at one level (e.g. the POA), with projections to several downstream sites evoking independent physiological responses. This pattern is seen in the projection from the VMPO to the DMH where activation of this pathway inhibits BAT thermogenesis but does not induce the additional vasodilation or cool environment preference seen when activating the VMPO itself ( Tan *et al*., 2016). It remains to be established why the DMH contains both excitatory and inhibitory neurons that drive thermogenesis ( Zhao *et al*., 2017), but a plausible explanation would be that it ensures tight or fail-safe control of this vital homeostatic process.

### Thermoregulation, food intake and body weight

There must be at least two processes that link food intake, thermoregulation, and maintenance of body weight. The first ensures that as changes in ambient temperature drive changes in energy expenditure, a commensurate adjustment of food intake occurs to maintain bodyweight. In this case, energy expenditure and food intake move together in parallel. Hence, cold exposure increases both BAT thermogenesis and food intake while warm exposure reduces both ( Kaiyala *et al*., 2012; Ravussin *et al*., 2014; Xiao *et al*., 2015). The second process is driven by the need to maintain a steady body weight. In this scenario, energy expenditure and food intake move in opposite directions: calorie-restriction drives food intake and suppresses body temperature, whereas calorie excess increases body temperature and suppresses food intake in humans ( Soare *et al*., 2011), as well as rodents ( Duffy *et al*., 1989; Rothwell & Stock, 1997).

**Figure 2.**
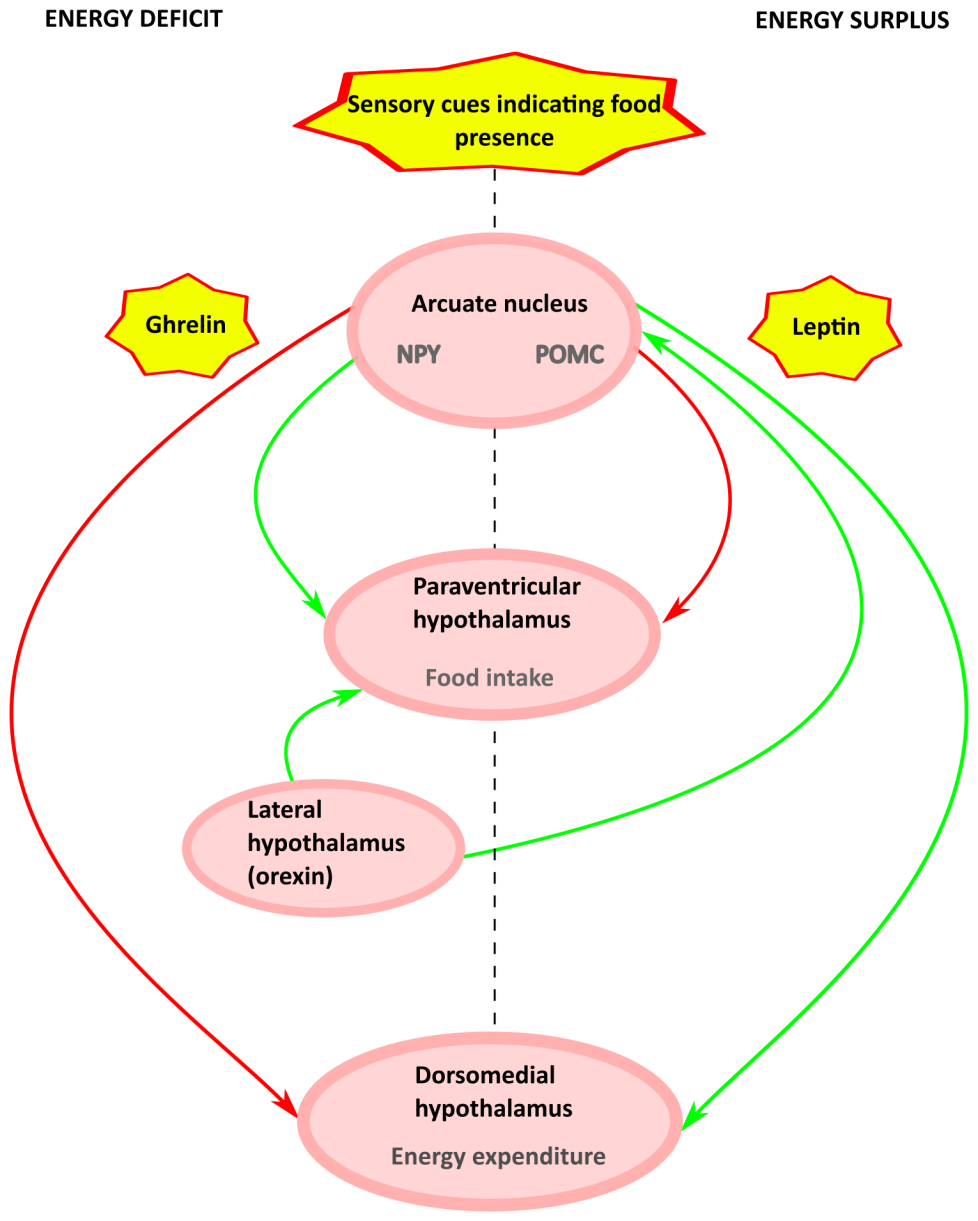
Schematic of the circuits controlling food intake and energy expenditure. Fasting stimulates the release of ghrelin from the gastrointestinal tract, which acts on arcuate NPY neurons. NPY-expressing neurons in the arcuate project to the PVH to stimulate food intake, and to the DMH to inhibit thermogenesis and reduce energy expenditure. Conversely, leptin, released from WAT in proportion to the amount of stored fat, acts on arcuate POMC neurons. Arcuate POMC neurons project to the PVH, where they inhibit food intake, and to the DMH, where they increase thermogenesis and energy expenditure. Orexin released from the lateral hypothalamus stimulates both food intake and thermogenesis. Sensory signals modulate the activity of arcuate NPY and POMC neurons in anticipation of food intake. At each level in the hypothalamus, the ghrelin-NPY circuits and the leptin-POMC circuits inhibit each other. Abbreviations: NPY, neuropeptide Y; PVH, paraventricular hypothalamic nucleus; DMH, dorsomedial hypothalamic nucleus; WAT, white adipose tissue; POMC, pro-opiomelanocortin;

The following section is not an exhaustive review of the regulation of appetite and food intake, but rather focuses on the mechanisms and anatomical regions in which the control of food intake interacts with thermoregulation and energy expenditure ( Figure 2). For a review of the central control of appetite, see here ( Andermann & Lowell, 2017).

Amongst several hypothalamic nuclei that contribute to control of food intake, the arcuate nucleus (ARC) is central and contains two distinct but intermingled populations of cells that perform opposing functions. One population responds to leptin, which is released from white adipose tissue (WAT) to signal satiety and replete WAT stores. These neurons express the neuropeptide pro-opiomelanocortin (POMC), which is cleaved into α-melanocyte stimulating hormone (α-MSH), which in turn acts on melanocortin receptors ( Cone, 2005; Dodd *et al*., 2015). The second population express neuropeptide Y and agouti-related peptide (NPY and AgRP, respectively). This group is activated by ghrelin, a hormone secreted by the gastrointestinal tract during fasting that stimulates food intake ( Hahn *et al*., 1998; Müller *et al*., 2015), and is inhibited by leptin. NPY/AgRP neurons, therefore, signal energy deficit and hunger. These two populations are mutually antagonistic: leptin depolarises ARC POMC neurons while hyperpolarising NPY/AgRP neurons, which reduces their inhibitory input onto the POMC population ( Cowley *et al*., 2001; Myers *et al*., 2008). Meanwhile, AgRP is a potent antagonist at melanocortin receptors (reviewed here ( Andermann & Lowell, 2017)). Each population and their interactions will be summarised below. 

### Leptin, pro-opiomelanocortin, and α-melanocyte stimulating hormone

Leptin is a peptide hormone released by WAT in proportion to the size of the adipose tissue reserve, reflecting long-term food intake and energy stores ( Frederich *et al*., 1995). In addition to reflecting WAT stores, leptin release is suppressed during periods of fasting in mice ( Swoap *et al*., 2006) and in humans ( Bergendahl *et al*., 2000). Leptin receptors are widespread, but of particular relevance here, are found in the ARC, PVH, DMH, POA, and the nucleus of the solitary tract (NTS) ( Myers *et al*., 2008; Zhang *et al*., 2011). Leptin release is also inhibited by exposure to a short photoperiod in hamsters ( Freeman *et al*., 2004), an observation that may account for the observation that humans eat more as winter approaches ( de Castro, 1991).

### Physiology of leptin

As a signal of replete WAT stores, leptin inhibits food intake and increases energy expenditure through BAT thermogenesis, growth, and reproductive behaviours ( Frederich *et al*., 1995; Myers *et al*., 2008; Schwartz *et al*., 1996). Exogenous leptin blunts the neuroendocrine consequences of starvation ( Ahima *et al*., 1996) and suppresses food intake ( Mistry *et al*., 1997). Leptin deficient ( *ob/ob*) mice are obese, due to both increased *ad-libitum* food intake ( Welton *et al*., 1973) and reduced basal metabolic rate, such that *ob/ob* mice pair-fed with lean littermates maintain a higher body mass with increased fat stores ( Trayhurn & James, 1978).

In keeping with a role linking energy balance and thermoregulation, leptin-deficient mice show defects in thermoregulation: hypothermic at sub-thermoneutral ambient temperatures and fatally incapable of defending body temperature with acute exposure to low ambient temperatures ( Kaiyala *et al*., 2015; Trayhurn *et al*., 1977). In addition to a central deficit in leptin signalling, *ob/*ob mice show reduced BAT response to electrical or noradrenergic stimulation, indicating a role for peripheral leptin in priming BAT thermogenesis ( Seydoux *et al*., 1982). Hence, the *ob/ob* mice are unable to recognise their ample fat stores and adapt as if in a starved state by suppressing thermogenesis and increasing food intake.

### Functional anatomy of leptin

Leptin receptors are highly-expressed in the ARC, which is a central hub in the processing of signals regarding the energy state of an animal ( Chen *et al*., 2015; Cone, 2005). Leptin’s actions here suppress food intake and, when combined with high circulating insulin, increase the sympathetically-mediated browning of WAT ( Dodd *et al*., 2015). Leptin’s effects in the ARC are mediated by a coordinated increase in the activity of POMC neurons, and reduction in the activity of neurons that co-express NPY and AgRP ( Cowley *et al*., 2001; Myers *et al*., 2008). POMC-expressing ARC neurons project to diverse brain regions including PVH, where they release α-MSH, which acts on melanocortin receptors ( Cone, 2005).

*In-*vivo calcium imaging reveals that the ARC POMC neurons react to both the presence of food and the sensory anticipation of food availability. This suggests that leptin-responsive POMC neurons have a role in acute satiety and foraging, in addition to long-term energy balance ( Chen *et al*., 2015). Transgenic mice lacking the melanocortin 4 receptor show a similar phenotype to *ob/ob* mice: obese with increased food intake and suppressed energy expenditure. Selective reintroduction of melanocortin 4 receptors in the PVH normalises the excess food intake but not the suppressed energy expenditure seen in these mice ( Balthasar *et al*., 2005).

In addition to running through circuits that modulate food intake, leptin receptors are also expressed in regions involved in thermoregulation: neurons that express leptin receptors are trans-synaptically labelled by pseudorabies virus injected into BAT. This delineates the presence of leptin receptors throughout the efferent thermoregulatory circuit from BAT, up to the RPa, the DMH, and finally, the median POA (MnPO). Leptin receptor-expressing neurons in the DMH produce c-Fos in response to acute cold exposure ( Zhang *et al*., 2011). Chemogenetic activation of these DMH leptin receptor-expressing neurons stimulates BAT thermogenesis and locomotor activity without affecting food intake, resulting in decreased body weight ( Rezai-Zadeh *et al*., 2014). Hence there is overlap between the circuits mediating the thermogenic effects of leptin and those mediating the thermogenic effect of acute cold exposure.

Neurons in the POA that express leptin receptors also respond to exposure to a warm environment. Chemogenetic activation of these neurons inhibits feeding and reduces energy expenditure by inhibiting BAT thermogenesis ( Yu *et al*., 2016). The simultaneous inhibition of feeding and thermogenesis is not usually a role attributed to leptin. Rather, leptin is usually associated with suppression of feeding and stimulation of thermogenesis ( Myers *et al*., 2008). Hence one might anticipate that the effect of leptin on these neurons would be to inhibit their activity. Indeed, the leptin receptor has been shown to be capable of inhibiting synaptic transmission ( Thompson & Borgland, 2013). These observations were dependent on ambient temperature, which suggests a role for POA leptin receptor-expressing neurons linking the necessary alterations of food intake that must accompany changes in the energy demands of thermogenesis. It would be interesting to establish the effects of leptin at its receptors in these neurons, and to identify their projection targets. 

In conclusion, the picture that emerges from the literature is that leptin tends to move energy expenditure and food intake in opposite directions: increased leptin drives weight loss by suppressing food intake and increasing energy expenditure. This suggests that its primary role is to control body mass and / or WAT stores. It may also contribute to suppressing both thermogenesis and food intake following exposure to a warm environment, although this is less well established.

### Neuropeptide Y and agouti-related peptide

NPY is one of the most highly expressed neuropeptides found in the brain, where it is commonly co-expressed with AgRP. It is involved in energy homeostasis, circadian rhythms, the stress response, and cognition. NPY production is widespread, but of particular relevance to energy homeostasis, it is expressed in the ARC, the NTS, the DMH, and the PVH ( Bi *et al*., 2003; Chronwall *et al*., 1985; Elmquist *et al*., 1999). There are three main NPY receptors in humans and rodents: Y1, Y2, and Y5. Expression of these receptors is also widespread, but densities occur in areas related to homeostasis, thermoregulation, and energy expenditure including: PVH, ARC, lateral hypothalamus, NTS and DMH. As introduced above, NPY and AgRP respond to fasting or WAT store depletion by suppressing energy expenditure and increasing food intake. Hence, NPY/AgRP have opposite and antagonistic functions to leptin (reviewed in Cone, 2005; Reichmann & Holzer, 2016).

### Physiology of NPY and AgRP

Peripheral administration of ghrelin induces c-Fos expression in, and depolarises, ARC NPY/AgRP neurons ( Chen *et al*., 2015; Cowley *et al*., 2003; Wang *et al*., 2002). These ARC NPY/AgRP neurons also express leptin receptors, the action of which reduces NPY and AgRP expression ( Wang *et al*., 1997), and causes hyperpolarisation ( Cowley *et al*., 2001). Hence, NPY and AgRP neurons are activated by low WAT energy stores and hunger. As with POMC neurons, NPY/AgRP neurons are modulated by the sensory anticipation of food, such that in food-restricted mice, presentation of sensory cues indicating food availability reduces firing ( Chen *et al*., 2015). Adult ablation of NPY/AgRP neurons leads to mice with low body weight, reduced food intake, and increased BAT activity ( Bewick *et al*., 2005; Gropp *et al*., 2005; Luquet *et al.*, 2005). Intra-cerebroventricular (ICV) administration of NPY acutely increases food intake and decreases BAT activity. ICV NPY also increases WAT lipoprotein lipase activity (indicating increased lipid deposition), an effect that persists in food-restricted rats, suggesting a direct effect rather than as a consequence of increased metabolic substrate availability ( Billington *et al*., 1991).

The effects of NPY on both BAT and WAT are mediated by sympathetic innervation, rather than via a circulating factor ( Egawa *et al*., 1991). NPY’s ability to drive lipoprotein lipase is an interesting observation since one might anticipate that NPY would liberate fat stores in an animal that is hungry. Instead, the activation of lipoprotein lipase suggests that the role of NPY is directed primarily towards replenishing fat stores rather than providing energy for immediate metabolism.

### Functional anatomy of NPY and AgRP

Local injections of NPY into discrete hypothalamic nuclei has at times produced contradictory findings. Injection into the ARC induces hypothermia, as might be expected ( Jolicoeur *et al*., 1995). NPY injection into the PVH may inhibit BAT sympathetic nerve activity ( Egawa *et al*., 1991), and yet it has also been shown to induce hyperthermia ( Jolicoeur *et al*., 1995). Likewise, injection into the medial preoptic area (MPA) can increase sympathetic nerve activity ( Egawa *et al*., 1991), but may also induce hypothermia ( Jolicoeur *et al*., 1995). This bidirectional response might indicate that high doses of NPY activate autoreceptors in a negative feedback loop to block transmission. This could result in increased BAT thermogenesis. This hypothesis is supported by the observation that ICV injection of NPY at low doses induces hypothermia and at higher doses causes hyperthermia ( Jolicoeur *et al*., 1995). Inhibition of NPY release by activation of presynaptic Y2 receptors has been observed *in-vitro* ( King *et al*., 1999). This is an important consideration with implications for experiments that use agonist injections or opto- or chemogenetics to activate circuits in a potentially supra-physiological manner.

ARC NPY /AgRP neurons project to the DMH and PVH, both of which also contain cell bodies that express NPY ( Chao *et al*., 2011; Chronwall *et al*., 1985; Tiesjema *et al*., 2007; Wang *et al*., 1997). While both acute and chronic food restriction induce NPY mRNA expression in the ARC, only chronic food-restriction induces NPY mRNA in the DMH ( Bi *et al*., 2003). Knock-down of DMH NPY expression increases WAT browning, lipolysis, and BAT UCP1. This increased BAT thermogenesis combined with observed increases in locomotor activity is not accompanied by increases in food intake, and therefore results in weight loss ( Chao *et al*., 2011). The effect of NPY knockdown in the DMH mimics the effect of leptin in this nucleus, with a tendency to affect thermogenesis and energy expenditure more than food intake ( Rezai-Zadeh *et al*., 2014). The DMH, however, may be capable of increasing food intake via a cholinergic input to the ARC, which increases inhibitory tone on ARC POMC neurons ( Jeong *et al*., 2017). One might speculate that this projection forms the basis for the link that drives increased food intake when the energetic costs of thermogenesis are high. Opto- and chemogenetic manipulation of ARC NPY/AgRP neurons that project to the PVH indicates that this circuit stimulates feeding via GABAergic input on PVH oxytocin neurons ( Atasoy *et al*., 2012; Chen *et al*., 2015).

### Food intake and thermoregulation: summary

The ARC, DMH, and PVH form a circuit that integrates information about the past, the present, and the future energy state. These signals are generated by leptin, ghrelin, and sensory inputs, respectively. Leptin signals long-term energy balance as reflected by WAT stores. Ghrelin signals recent food intake and time since last meal. Sensory inputs signal the approaching likelihood of food. The system comprises parallel antagonistic and mutually inhibitory branches: elevated leptin indicates replete energy stores and releases the brakes on energy expenditure while inhibiting further food intake by activating POMC neurons and inhibiting ARGP/NPY neurons; suppressed leptin and/or elevated ghrelin signals depletion of energy stores, driving food intake and suppression of energy expenditure through activation of NPY/AgRP and inhibition of POMC neurons.

At each level neurons that are activated by ghrelin are generally inhibited by leptin and vice versa. A model that emerges from review of the literature is that the ARC to PVH projection responds rapidly to cues regarding acute energy requirements and food availability to drive changes in food intake. On the other hand, the ARC to DMH projection appears more concerned with adjusting energy expenditure so that WAT stores are maintained within a target range. Within this putative framework, the PVH might modulate feeding through the NTS, while the DMH would modulate energy expenditure via the RPa.

### Thermoregulation and sleep

In humans intracranial temperature reaches a peak in the period one to two hours prior to the onset of darkness, and drops by approximately 1°C during sleep ( Landolt *et al*., 1995). The cooling associated with sleep is an active process, in humans driven at least in part by peripheral vasodilation and heat dissipation through sweating ( Kräuchi *et al*., 2000). Similar alterations in core and brain temperature during sleep are seen in rodents ( Harding *et al*., 2019; Zhang *et al*., 2015).

Non rapid-eye-movement (NREM) sleep is characterised by a controlled reduction in body temperature such that while core body temperature reduces, changes in ambient or hypothalamic temperature continue to induce thermoregulatory responses, including sweating ( Geschickter *et al*., 1966), panting, and shivering ( Parmeggiani & Rabini, 1967). During NREM sleep, the temperature threshold for inducing metabolic heating is reduced, and the slope of the response also reduced compared to wake ( Glotzbach & Heller, 1976). In contrast, rapid-eye-movement (REM) sleep appears to involve total cessation of thermoregulation such that (in small animals at least) body temperature follows changes in ambient temperature, and does not respond to changes in local hypothalamic temperature by increasing metabolic heat production ( Glotzbach & Heller, 1976; Heller & Glotzbach, 1977; Walker *et al*., 1983). This abandonment of thermoregulation during REM may be a factor limiting the duration of REM epochs in sub-thermoneutral environments ( Heller & Glotzbach, 1977). REM sleep is also characterised by cerebral oxygen consumption similar to that seen during waking, which is in stark contrast with NREM sleep ( Madsen *et al*., 1991).

The interaction between sleep and body temperature is reciprocal: sleep is associated with a reduction in body temperature, and increased body or hypothalamus temperature or a warm ambient environment promotes sleep ( Bunnell *et al*., 1988; Kräuchi *et al*., 1999; Lo Martire *et al*., 2012; McGinty & Szymusiak, 1990; Romeijn *et al*., 2012). This relationship has led some to hypothesise that sleep is fundamentally a thermoregulatory homeostatic process ( McGinty & Szymusiak, 1990). The observation that vasodilation and a rapid rate of body cooling is associated with sleep onset appears somewhat at odds with the fact that prior to sleep humans and rodents seek out warmth ( Harding *et al*., 2018). The reason behind this apparent paradox may lie in the observations that while increased temperature, be that core, brain, or ambient is associated with increased sleep, in particular NREM sleep, it is not the elevated temperature but rather the subsequent high rate of heat loss that seems to be most predictive of sleep initiation.

In humans, increasing peripheral vasodilation associated with a falling core body temperature from its peak in the hours prior to sleep onset predicts latency to sleep ( Kräuchi *et al*., 1999; Kräuchi *et al*., 2000). Humans also tend to select a bed time that coincides with the maximum rate of circadian body temperature reduction ( Campbell & Broughton, 1994). A similar observation has been made in mice: reactivating warm-sensing POA neurons induces a drop in core temperature and increased NREM sleep ( Harding *et al*., 2018). The tendency to seek warm environments prior to sleep may therefore help to activate these sleep-inducing POA neurons.

On the other hand, the high rate of heat loss and relative unresponsiveness of the thermoregulatory system may reflect an undesirable but inevitable side-effect of the process of sleep induction. In this case, seeking out warm environments prior to sleep would be a means to mitigate some of the heat loss that results from those side-effects. For example, if reduced sympathetic tone is required to allow the animal to enter a low vigilance state prior to sleep onset, then a corollary of that might be increased vasodilation and reduced BAT thermogenesis. 

### Functional anatomy linking sleep and thermoregulation: the preoptic hypothalamus

The POA acts as a critical hub linking thermoregulation and sleep. In mice, recovery sleep following a period of deprivation is associated with a drop in core temperature of between 1.5 and 2°C, and increased delta power, indicating NREM sleep. Chemogenetic reactivation of median and lateral POA neurons that are active during recovery sleep recapitulates both this increased NREM sleep and drop in core body temperature. Lateral POA (LPO) neurons are also the target of the α-2 agonist dexmedetomidine, which induces sedation that mimics recovery sleep ( Kroeger *et al*., 2018; Zhang *et al*., 2015).

The population of neurons in the region of the LPO and VLPO that are capable of inducing NREM and hypothermia express galanin: chemo- or optogenetic stimulation of GABA- and galanin-expressing neurons in the ventrolateral preoptic nucleus (VLPO) induces NREM and a drop in body temperature ( Kroeger *et al*., 2018); knock-out of lateral preoptic (LPO) galanin neurons significantly attenuates the sedation and hypothermia associated with dexmedetomidine administration, and causes a rise in body temperature with disrupted sleep homeostasis ( Ma *et al*., 2019).

These findings support the hypothesis that reduced sympathetic tone associated with activation of presynaptic α-2 receptors disinhibits sleep- and hypothermia-promoting galanin neurons in the lateral and/or ventrolateral preoptic area. VLPO neurons that are active during sleep project monosynaptically to the tuberomammillary nucleus, which is known to modulate arousal ( Sherin *et al*., 1996). In a related study, warm sensitive neurons in the region of the MnPO/MPO were reactivated using activity-dependent tagging. While reactivation of a GABAergic subpopulation of these neurons induced NREM sleep without a significant change in body temperature, reactivation of the glutamatergic/nitrergic subpopulation induced both, indicating that they may be part of the circuit that coordinates NREM sleep induction with body temperature reduction ( Harding *et al*., 2018).

In all these experiments, the drop in body temperature during chemogenetic-driven sleep was deeper than that seen in natural sleep ( Harding *et al*., 2018; Kroeger *et al*., 2018; Zhang *et al*., 2015). This observation may reflect the somewhat abnormal nature of the stimulation ( Armbruster *et al*., 2007), or may reflect an additional role for these regions in torpor.

### Sleep and adenosine

The role of adenosine in sleep is complex and beyond the scope of this review; presented here is a summary of some key aspects, as they relate to torpor (a more comprehensive review is provided here ( Silvani *et al*., 2018)). The G protein-coupled adenosine receptors A1R, and A2R are widely expressed throughout the brain. The A1R is inhibitory and generally considered to be neuroprotective through suppression of glutamate release and hyperpolarisation (reviewed here ( Cunha, 2005)), and by modulating cerebral blood flow and metabolic rate ( Blood *et al*., 2003). In addition to their central effects, adenosine receptors in the cardiovascular system mediate negative inotropic, chronotropic, dromotropic, and anti-adrenergic effects via A1Rs, and vasodilatation via A2Rs (reviewed here ( Shryock & Belardinelli, 1997)). Central activation of A1Rs promotes sleep, hypothermia, sedation, and reduced locomotor activity ( Anderson *et al*., 1994).

Sleep deprivation increases the homeostatic drive for sleep, and is reflected in elevated time spent in NREM and by increased EEG delta power during subsequent recovery sleep (reviewed here ( Borbély *et al*., 2016)). Expression of this rebound increase in NREM sleep is dependent on the presence of neuronal A1Rs, via an interaction with glia ( Bjorness *et al*., 2009; Bjorness *et al*., 2016), although additional mechanisms may also be capable of providing this function, for example in whole-animal A1R knockouts ( Stenberg *et al*., 2003). In this way, adenosine links the homeostatic drive for sleep with suppression of metabolic and cardiovascular systems, and induction of NREM sleep.

### Sleep and torpor

Preserved thermoregulatory control despite altered body temperature is characteristic of both torpor and NREM sleep. Both also probably reduce overall energy expenditure. An obvious question is to ask what is the link between torpor and NREM sleep?

Ground squirrels and pocket mice enter torpor through sleep ( Berger, 1984; Heller & Glotzbach, 1977; Walker *et al*., 1977). Electroencephalogram (EEG) recordings during torpor display the characteristics of NREM sleep provided brain temperature is above about 25°C. At brain temperatures below 25°C, EEG power is globally reduced but delta waves associated with NREM sleep are discernible. EEG power decreases (and with it, the ability to discern sleep states) with brain temperatures below 20°C, and becomes isoelectric below about 10°C ( Larkin & Heller, 1996; Walker *et al*., 1981).

Consistent with the observation that low brain temperatures are associated with the loss of NREM EEG pattern, there is evidence that prolonged torpor such as that seen in seasonal hibernators is associated with accumulation of sleep debt. During prolonged seasonal hibernation periods, arctic ground squirrels periodically arouse to euthermia through NREM sleep. The duration of the post-arousal NREM sleep correlates with the minimum brain temperature reached during the preceding torpor ( Larkin & Heller, 1996; Trachsel *et al*., 1991). These observations indicate that while torpor at intermediate core temperatures resembles NREM sleep, some of the vital functions of sleep are depressed during torpor at very low body temperature, and must be performed at or close to euthermia.

## Efferent signals triggering torpor entry

### The sympathetic nervous system and leptin in torpor

Dopamine beta-hydroxylase (DBH) knock-out mice (DBH-/-) lack norepinephrine and epinephrine, while their heterozygous littermates appear essentially normal. Norepinephrine can be at least partially restored by the administration of L-threo-3,4-dihydroxyphenyserine (DOPS) ( Thomas *et al*., 1995; Thomas *et al*., 1998). DBH-/- mice fail to enter torpor after 12 hours of fasting at 20°C. This impairment in torpor can be reversed by the administration of DOPS to restore adrenergic signalling, or by selective activation of beta-3 adrenoceptors. Serum leptin is elevated in both the fed and fasting state in DBH-/- compared to DBH+/- mice. Fasting does not significantly reduce serum leptin in DBH-/- mice, but fasting in combination with administration of DOPS or a beta-3 agonist reduces serum leptin to levels comparable to fasted DBH+/- mice ( Swoap *et al*., 2006).

The model that emerges from this series of experiments is that activation of beta-3 receptors on WAT suppresses leptin release, which serves as a signal for torpor induction. There are additional studies that support this model. Firstly, DBH-/- mice that also lack leptin signalling (by crossing with *ob/ob* mice to generate double-mutant mice) regain the ability to enter torpor, albeit displaying unusually early and shallow bouts. The proposal is that in lacking leptin, these modifications bypass the need for sympathetic action on WAT. Once torpid, these double knock-out mice are unsurprisingly slow to rouse given they lack both leptin, which is BAT thermogenic, and norepinephrine, which acts on beta-3 receptors in BAT to stimulate thermogenesis ( Swoap & Weinshenker, 2008). Secondly, exogenous leptin reduces leptin mRNA expression in WAT of DBH+/- mice but does not suppress expression in DBH-/- WAT, indicating that the autoregulation of leptin is dependent on norepinephrine (or perhaps epinephrine) ( Commins *et al*., 1999). Thirdly, torpor in short photoperiod-adjusted Djungarian hamsters can be blocked by chemical sympathectomy with 6-Hydroxydopamine ( Braulke & Heldmaier, 2010).

This is an appealing model, but there are some caveats:

1.   While the torpor bouts generated by administration of DOPS to fasted DBH-/- mice appeared similar to those seen in DBH+/- controls, administration of a beta-3 agonist (CL 316243) produced a hypothermia so profound that the animals did not spontaneously arouse ( Swoap *et al*., 2006). It is not entirely clear, then, that this was the same as natural torpor.

2.   If activation of beta-3 receptors on WAT serves as the first step towards torpor induction, then administration of a beta-3 agonist to wild type mice should increase the probability and or depth of torpor: this has not been reported.

3.   Fasted DBH+/- mice given a selective beta-3 receptor antagonist appear to enter torpor normally, with a rate of decline in core body temperature that is comparable to controls ( Swoap *et al*., 2006). The difference between this and control torpor bouts appears to be that the beta-3 receptor antagonist caused the torpor bout to be terminated, before core temperature reaches a ‘normal’ nadir. This does not fit with the model that beta-3 receptor suppression of WAT leptin release is the initiating trigger for torpor.

4.   Given that leptin acts on POMC/α-MSH neurons in the arcuate, blocking this pathway should mimic a drop in leptin and therefore be pro-torpor. However Ay mice, which display ectopic AgRP production and through the antagonist effect of AgRP on melanocortin 4 receptors, impaired α-MSH – melanocortin signalling, in fact show a reduced tendency to torpor ( Gluck *et al*., 2006).

Although there is a correlation between the ability to suppress leptin and the ability to enter torpor, a causal nature for this relationship has not been exhaustively demonstrated. An implication of this model is that exogenous leptin should prevent torpor, and that interfering with leptin signalling should induce torpor even in a fed state. These have been difficult to demonstrate, and will be discussed in more detail below.

Mice lacking leptin, the *ob/ob* mice, are prone to deeper and longer torpor bouts than wild type (WT) mice on fasting or food restriction, despite their large adipose tissue stores ( Gavrilova *et al*., 1999; Himms-Hagen, 1985). However, it is worth pointing out that the *ob/ob* mouse is not permanently torpid, and neither are A-ZIP/F-1 mice, which have both dramatically reduced WAT and BAT and persistently low leptin levels. While A-ZIP/F-1 mice will readily enter torpor on fasting, exogenous leptin administration does not prevent fasting-induced torpor in these mice. In contrast to this, leptin administration to *ob/ob* mice may block torpor entry ( Gavrilova *et al*., 1999). Interpreting the effects of leptin administration to transgenic mice that have adapted to absent leptin signalling is challenging, especially given that the expression of torpor in these mice, even without the additional complexity of adding exogenous leptin, is not the same as torpor seen in WT mice.

Studying the effect of leptin on torpor in WT mice has also produced contradictory findings. One study reports no effect of leptin treatment on core temperature of WT mice during a 24 hour fast ( Gavrilova *et al*., 1999). In this study leptin administration to male WT mice fasted for 24 hours did not prevent the drop in core temperature seen in saline-treated mice. However, while the core temperature of both leptin-treated and control male WT mice during 24 hours of fasting did decrease, it remained above 30°C, and therefore above commonly accepted thresholds for torpor. Hence, while leptin prevented any core temperature response to fasting in *ob/ob* mice, it had no effect on WT mice. Why leptin might abolish torpor in *ob/ob* mice but not affect temperature responses to fasting in WT mice is unclear, but may reflect differences in the sensitivity of *ob/ob* mice to exogenous leptin.

In another study, male WT mice fasted for up to 48 hours showed fasting-induced suppression of metabolism and core temperature, but not full torpor. Leptin treatment reduced fasting-induced hypometabolism in these WT mice ( Bechtold *et al*., 2012). The reasons for these different results are not clear, but may reflect differences in the administration of leptin: in the former study, in ( Gavrilova *et al*., 1999), leptin was administered via continuous subcutaneous (SC) infusion whereas in the latter study ( Bechtold *et al*., 2012), leptin was delivered in a single ICV injection. Whatever the reason for these differing results, neither have confirmed that leptin delivery to WT mice prevents full torpor bouts.

Leptin treatment in fasted marsupial mammal ( *Sminthopsis macroura*) reduces the duration and depth of daily torpor bouts ( Geiser *et al*., 1998), but again the effect of leptin in this species appears to be predominantly to impair the maintenance rather than the initiation of torpor. It seems that exogenous leptin might reduce the probability of torpor entry in Siberian hamsters although in those leptin-treated hamsters that did enter torpor, the torpor bout depth, duration, and frequency remained comparable to torpor bouts in control hamsters. Comparing hamsters housed under identical conditions that did or did not enter torpor revealed no difference in endogenous serum leptin levels. Likewise, the serum leptin levels were the same in individual animals on days in which the animal did or did not enter torpor. Finally, while animals that entered torpor tended to have low leptin, the lowest levels were recorded in hamsters that did not enter torpor ( Freeman *et al*., 2004).

In summary, the evidence for leptin’s role in torpor garnered from transgenic models varies depending on whether the model used is the primarily leptin-deficient *ob/ob* line, or the A-ZIP/F-1 line in which absent leptin is secondary to persistently depleted adipose tissue stores. While both models result in low leptin and increased propensity to torpor, only the *ob/ob* mice are sensitive to leptin replacement. That mice from neither line are in a permanent state of torpor would suggest either adaptive mechanisms appear during development, or else a permissive rather than a sufficient role of low leptin in torpor. Attempts to establish the effects of leptin administration to WT mice have been hampered by the fact that the WT control mice in these experiments were not entering full torpor. That said, converging evidence both from studies specifically examining leptin and torpor, as well as studies looking at the role of leptin under more ‘normal’ physiological settings, indicates that it is likely that high leptin would inhibit torpor and conversely low leptin likely forms at least part of the signal for torpor. Finally, evaluation of the studies to date raises the possibility that the beta-3 adrenoceptor-driven suppression of leptin plays a greater role in maintaining than initiating torpor.

### NPY, ghrelin, and torpor

Since ghrelin and NPY act as the counterbalance to leptin, signalling hunger and energy deficit (see section 1.3.4), it is reasonable to hypothesise that they contribute to the signal for torpor. Ghrelin injection during a fast in a cool ambient temperature deepens and prolongs torpor bouts in mice but does not induce torpor in the fed state. NPY-/- mice exhibit shallow and aborted torpor bouts, which are not rescued by peripheral ghrelin. This indicates that ghrelin exerts its effects on torpor via NPY neurons ( Gluck *et al*., 2006).

ICV injection of NPY in cold-acclimated Siberian hamsters (small, heterothermic mammals) reduces core body temperature and can increase the probability of torpor. This effect in hamsters is mediated by Y1 receptors ( Dark & Pelz, 2008). ICV NPY may also inhibit food intake, in proportion to its effects on body temperature or torpor ( Paul *et al*., 2005). This latter finding is surprising given NPY is usually considered orexigenic. At some point, in order to enter torpor, the normal response to hunger, which is to forage and increase food intake, is presumably switched to a signal to cease locomotor activity and enter torpor; perhaps this observation reflects that transition. It is also relevant to note that hamsters undergo both fasting-induced torpor, which is triggered by energy deficit at any seasonal time, and short photoperiod-induced torpor, which is seasonal and does not necessarily involve an energy deficit. These distinct torpor phenotypes may involve different regulatory mechanisms ( Cubuk *et al*., 2017), which could account for the observed effect of NPY on food intake in these animals.

The arcuate is a key locus for NPY signalling, and selective ablation of ARC neurons with monosodium glutamate (MSG), supports a role for this nucleus in torpor. For example, in contrast to controls, ARC-ablated mice do not enter torpor after 24 hours of fasting, although they do show a degree of fasting-induced hypothermia ( Gluck *et al*., 2006). In Siberian hamsters, ARC ablation impairs short photoperiod-induced torpor, reducing the probability, and slightly reducing the depth and length of torpor bouts. However, torpor was still seen in these hamsters and there was no difference in residual ARC NPY immunoreactivity between ARC-ablated hamsters that did and those that did not enter torpor. Likewise, ARC ablation reduced the probability of torpor in food restricted hamsters but had no effect on the quality or frequency of those torpor bouts in animals in which torpor was seen.

Although NPY receptor antagonists have been shown to prevent NPY-induced hypothermia ( Dark & Pelz, 2008), the same has not been demonstrated for natural torpor. This raises questions about whether the hypothermia seen following NPY injection is torpor, or rather an exaggerated form of the starvation-induced drop in temperature that is seen in non-hibernators ( Billington *et al*., 1991), although of course the two may lie on a continuum.

In summary, there is evidence indicating roles for ghrelin and NPY within the ARC as signals for the conditions that are associated with torpor. There is also some evidence supporting direct roles in torpor, and a functioning ARC nucleus may be a requisite for the expression of torpor in mice. However, this necessity has not been demonstrated in hamsters, indicating either that alternative mechanisms might exist, capable of bypassing the ARC, or else suggesting that torpor in hamsters and mice is generated through distinct mechanisms. To date, there is no evidence that activity of ARC neurons is sufficient to induce a torpor bout.

### Adenosine, orexin, and torpor

Adenosine, which was introduced above, is a natural candidate to link many of the functions associated with torpor ( Silvani *et al*., 2018). Central infusion of the A1R agonist N ^6^-cyclohexyladenosine (CHA) into rats exposed to cold ambient temperature generates a state that has many features of torpor, including vagally mediated skipped beats and bradycardia, inhibition of BAT and shivering thermogenesis, and decreased EEG power ( Tupone *et al*., 2013). Accumulation of adenosine during periods when demands for ATP outstrip supply, and the consequent engagement of a repertoire of responses that limit ATP consumption (reviewed here ( Newby, 1984)), make it an appealing candidate for signalling the drive for torpor.

Prolonged subcutaneous infusion of aminophylline, a non-specific adenosine receptor antagonist, significantly impairs torpor in male mice, resulting in delayed, shallow, and brief torpor bouts. Aminophylline infusion initiated during torpor triggers emergence ( Iliff & Swoap, 2012). In animals that enter torpor in response to seasonal cues, the response to adenosine is dependent on those cues. For example, central A1R blockade in Syrian hamsters causes arousal during the induction phase of seasonal torpor ( Tamura *et al*., 2005). In arctic ground squirrels, ICV infusion of CHA induces torpor or a similar state, in a manner that was modulated by the season, and was blocked by central A1R antagonists ( Jinka *et al*., 2011). Calorie restriction by alternate day feeding suppresses core temperature and respiratory rate in rats, and increases the sensitivity to CHA by increasing the expression of A1Rs in the hypothalamus ( Jinka *et al*., 2010). Hence, modulation of the central sensitivity to adenosine provides a means for both hibernators and non-hibernators to adjust temperature responses to environmental cues.

Despite the striking similarities between torpor and the physiological response to central A1R activation, there are some features that remain distinct. Changes in heart rate with torpor occur rapidly and display frequent skipped beats and asystoles, whereas those changes occur over several hours following CHA treatment and involve predominant extension of the inter-beat interval and rarely display asystoles. Temperature changes are slower in natural torpor compared to CHA-driven hypothermia, with no evidence of shivering in the latter. Finally, c-Fos is induced in the liver and heart of mice treated with CHA, but not in natural torpor, indicating calcium influx and potentially signalling cellular stress following CHA treatment ( Vicent *et al*., 2017a). Perhaps most importantly, fasting-induced torpor persists in mice lacking AR1 and AR3 ( Carlin *et al*., 2017).

Orexinergic neurons may mediate some of the thermoregulatory adaptations seen following central adenosine administration, since orexin -/- mice are less sensitive to the effects of central CHA administration. However, these same mice also recover more slowly from the hypothermia induced by CHA, and are prone to deeper, longer, and more frequent torpor bouts than WT controls. *In-vivo* calcium imaging in this study indicated that orexin neurons are active immediately prior to and after fasting-induced torpor ( Futatsuki *et al*., 2018). It is interesting to note that the interaction between orexin and CHA appears to be bidirectional: orexin enhances CHA-induced hypothermia initiation and overcomes it during recovery. Likewise, the effect of orexin on body temperature appears to depend on the sleep/wake cycle: promoting thermogenesis during waking and heat loss during sleep ( Mochizuki *et al*., 2006).

In summary, adenosine represents a candidate signal for torpor initiation but, once again, must be designated as ‘contributing’ or ‘permissive’ and not a necessary and sufficient master switch. One might expect orexin to reduce the likelihood of torpor, and to assist in arousals, and while this role is supported by the observation of increased torpor depth and duration in orexin -/- mice, the role in WT mice or other species is not clear. There is currently no accepted explanation for the apparent bidirectional effects of orexin on body temperature and following CHA administration.

### Torpor and endogenous opioids

The endogenous opioid system contributes to pain modulation, reward, the stress response, and several autonomic functions including digestion, arousal, and control of heart and respiratory rate. It comprises three groups of peptide transmitters: β-endorphin, enkephalins, and dynorphins, which act predominantly but not exclusively at µ-, δ-, and κ- opioid receptors, respectively. Of note, β-endorphin is produced in POMC neurons, by an alternate cleaving of the precursor POMC (reviewed here ( Benarroch, 2012)). Early investigations into the thermoregulatory effects of intracerebral β-endorphin injection reported that the effects depended on both the location of the injection and the dose used. For example, injection into the POA, anterior hypothalamus (AH), periaqueductal grey (PAG), nucleus accumbens (NAcc), reliably produced an initial hypothermia, with core temperature dropping by approximately 1°C. This was generally followed by a period of hyperthermia, except when high doses were injected into the NAcc, where high doses appeared to produce a sustained hypothermia ( Tseng *et al*., 1980). A similar effect is seen following administration of morphine to rats at increasing doses. These biphasic responses might be the result of time- and dose- dependent activation of different opioid receptor classes. Studying the effects of various opioid receptor-specific agonists and antagonists in rats and mice suggests that activation of κ- or δ-opioid receptors results in hypothermia, whereas the µ-opioid receptor mediates hyperthermia (reviewed here ( Rawls & Benamar, 2011)).

Some have argued for the existence of a ‘hibernation induction trigger’ (HIT) that circulates in blood of seasonal hibernators, and can be transfused from a hibernating individual into a non-hibernating individual with the effect of inducing hibernation ( Dawe & Spurrier, 1969), although this is controversial ( Wang *et al*., 1988). The apparent induction of hibernation via HIT transfusion is impaired by infusion of µ or κ agonists, whereas infusion of the δ agonist DADLE ([D-Ala, D-Leu]-Enkephalin) appeared to mimic the effects of HIT infusion by inducing hibernation in summer-active ground squirrels. It has therefore been argued that natural hibernation generates a circulating δ-receptor agonist that is capable of triggering hibernation ( Oeltgen *et al*., 1988).

Less controversial observations of the role of the endogenous opioid system in torpor derive from experiments infusing agonists or antagonists either locally or ICV in hibernating hamsters. Arousal from the maintenance but not the induction phase of torpor can be triggered by ICV naloxonazine (a µ1 opioid receptor antagonist) in hibernating Syrian hamsters ( Tamura *et al*., 2005). Thus maintained suppression of body temperature may depend on POMC neurons in the ARC that project to regions including DMH, AH, posterior hypothalamus (PH), and ventromedial hypothalamus (VMH) ( Tamura *et al*., 2012).

In summary, there is conflicting evidence from these experiments. In rats, and non-torpid mice, the evidence suggests that δ-opioid receptor activation induces hypothermia whereas µ-opioid receptors induce hyperthermia. One might therefore expect δ-opioid receptor activation to be involved in inducing or maintaining torpor. Experiments using HIT infusion to induce torpor or a torpor-like state in ground squirrels support this model, with a role for δ-opioid receptor activation in torpor induction, while µ- and κ-opioid receptors appear to inhibit torpor entry. In contrast to this, and out of keeping with the findings in rats and non-torpid mice, in Syrian hamster undergoing seasonal hibernation, the evidence would suggest that POMC neurons in the ARC activate µ-opioid receptors in several hypothalamic areas to maintain low body temperature in seasonal hibernation. It is difficult to draw any synergy from these findings: it is possible that different opioid receptors are involved in both promoting and inhibiting torpor, perhaps as part of a system that prevents excessively long or deep torpor bouts. Alternatively, it is worth considering whether the doses of agonists and antagonists used resulted in non-specific activation of several opioid receptor subtypes. It would be interesting to test the effects of modulating endogenous opioid pathways in mice undergoing daily torpor, as the data above only describes effects on seasonal hibernators or euthermic mice and rats.

### Neural control of torpor

In the 13-lined ground squirrel, a seasonal hibernator, *in-situ* hybridisation (ISH) reveals distinct patterns of c-fos expression during different phases of the hibernation cycle ( Bratincsák *et al*., 2007). Entrance into torpor is associated with increased c-fos mRNA in the ventrolateral part of the MPA, whereas arousal from torpor is associated with increased expression in the ventromedial part of the MPA. In awake animals during interbout arousals, the ARC and dorsolateral hypothalamus were active. The SCN and reticular thalamus were active throughout all stages of torpor, areas involved in circadian rhythm generation and inhibition of motor activity, respectively. In torpid mice the combination of c-Fos immunohistochemistry and retrograde tracer expression identifies a group of neurons in the DMH that project to the RPa, which are specifically activated during torpor ( Hitrec *et al*., 2019). It is anticipated that activating this pathway would inhibit thermogenesis by reducing the output from RPa to BAT, and indeed pharmacological inhibition of the rostral ventromedial medulla (a region that includes the RPa) induces a torpor-like state in the rat ( Cerri *et al*., 2013).

Three recent publications significantly furthered our understanding of the neural control of torpor entry, all converging on the preoptic area of the mouse hypothalamus as a key region, potentially containing neurons that represent a torpor ‘master switch’. One of these studies ( Hrvatin *et al*., 2020) used activity-dependent recombination (‘TRAPing’, ( Allen *et al*., 2017; DeNardo *et al*., 2019)) to selectively express designer receptors exclusively activated by designer drugs (DREADDs ( Alexander *et al*., 2009)) in neurons that were active during daily torpor in the mouse. Chemogenetic reactivation of neurons within the anterior and ventral portions of the medial and lateral preoptic area (avMLPA) generated a profound hypothermia and reduction in locomotor activity that appeared to mimic torpor.

These torpor-TRAPed avMLPA neurons project to several regions likely to be involved in torpor including the dorsomedial hypothalamus. Further experiments suggested that within the population of TRAPed preoptic area neurons, a subset of glutamatergic, PACAP expressing neurons generate the drop in temperature and activity observed during both natural and synthetic torpor. Blocking synaptic transmission in either glutamatergic or PACAP expressing neurons within the avMLPA impaired the expression of natural fasting-induced torpor ( Hrvatin *et al*., 2020).

The second paper, from the Sakurai group in Japan ( Takahashi *et al*., 2020), used a different approach but came to similar conclusions. They targeted expression of excitatory DREADDs to hypothalamic neuropeptide pyroglutamylated RFamide peptide (Qrfp) neurons. This neuropeptide was previously implicated in the modulation of food intake, adrenal activity, and anxiety, but not torpor ( Okamoto *et al*., 2016; Takayasu *et al*., 2006). Chemoactivation of Qrfp-expressing neurons in the medial preoptic area (MPA) and the anteroventral periventricular nucleus (AVPe, together termed AVPe/MPA) induced a long-lasting torpor-like hypothermic state in mice, with suppressed core temperature, oxygen consumption, heart rate, respiratory rate, and locomotor activity (termed QIH, for Q-neuron-induced hypothermia and hypometabolism).

QIH was recapitulated by selective optogenetic activation of the terminals of AVPe/MPA Qrfp neurons in the DMH. It was predominantly dependent on glutamatergic transmission within the Qrfp neurons population, although GABAergic Qrfp neurons appear to contribute to a smaller extent. Blocking synaptic transmission in Qrfp neurons impaired normal fasting-induced torpor resulting in a more gradual reduction in body temperature on fasting, and reduced the normal diurnal fluctuation in body temperature.

Hence, the authors identified a population of Qrfp-expressing neurons whose cell bodies lie in the preoptic area, which appear to have a role in generating the usual rapid decrease in core temperature associated with torpor induction, and whose terminals project to the DMH. Activation of this Qrfp neuron projection from the AVPe/MPA to the DMH generates a torpor-like state in mice. RNA in-situ hybridisation revealed that approximately 80% of Qrfp neurons also express PACAP, indicating significant overlap with the torpor-induing neurons identified by Hrvatin *et al.*

It is worth noting that Takahashi *et al.* did not demonstrate that the Qrfp neurons were active during torpor. Indeed, there were some differences between natural torpor and QIH induced by Takahashi *et al*., which appear to relate to whether the mouse is attempting to lose heat to the environment. During natural torpor, the mouse adopts a curled-up posture consistent with attempts to conserve heat, irrespective of the ambient temperature. During QIH at high ambient temperature, the mouse adopts an extended posture, consistent with attempts to lose heat. In addition, at 21°C ambient temperature, QIH is associated with an initial increase in tail surface temperature, indicating vasodilatation. In contrast, natural torpor may be associated with increased total peripheral resistance, which suggests at least on the whole-body scale, vasoconstriction ( Swoap & Gutilla, 2009). Hence, there remains a question regarding the degree of overlap between QIH and natural torpor.

Furthermore, Takahashi *et al.* did not confirm that the Qrfp neurons projecting from POA to DMH were glutamatergic or GABAergic. This is an important question, since the observed hypothermia might, for example, be the result of activating the established POA to DMH GABAergic projection that is involved in warm-sensing and thermoregulatory homeostasis ( Tan *et al*., 2016), rather than a specific torpor-inducing pathway.

Finally, Zhang *et al.* ( Zhang *et al*., 2020) demonstrated a population of oestrogen-sensitive neurons within the medial preoptic area whose activity increases during fasting-induced torpor in the mouse. Selective ablation of these neurons using Cre-dependent Caspase 3 expression impaired fasting induced torpor in these mice, suggesting a role in natural torpor. Chemogenetic activation of these neurons generated hypothermia and bradycardia as well as suppressed locomotor activity and oxygen consumption: all features of natural daily torpor in the mouse. However, they too noted peripheral tail vasodilatation associated with this DREADD-driven torpor-like state. Importantly, these oestrogen sensitive MPA neurons did not appear to respond to increased ambient temperature, indicating that they are not part of the thermal defence circuit ( Morrison & Nakamura, 2019).

### Towards a torpor circuit

The data presented in the studies of Hrvatin, Takahashi, and Zhang represent significant advances in our understanding of torpor. From this data, a model that emerges is that glutamatergic neurons in the preoptic area, which express PACAP and/or Qrfp, and perhaps oestrogen receptors, generate torpor. The preoptic area is well-placed for the role attributed to it in this model. It is a key site involved in thermoregulation and energy balance, receiving information regarding the external environmental temperature as well as directly sensing hypothalamic temperature ( Song *et al*., 2016), this information is then used to modulate BAT thermogenesis ( Tan *et al*., 2016; Zhao *et al*., 2017).

Warm-sensing glutamatergic POA neurons also play a role in coordinating the parallel decrease in core temperature observed with the onset of NREM sleep ( Harding *et al*., 2018). NREM sleep has several characteristics in common with torpor: it is a hypoactive, hypometabolic, bradycardic state, with maintained thermoregulation despite a reduced core body temperature ( Glotzbach & Heller, 1976; Heller & Glotzbach, 1977; Kräuchi, 2007; Schwimmer *et al*., 2010). Supposing that torpor is induced by the same circuit that links reduced body temperature with NREM sleep onset, then the distinction between the two states might rest upon the degree to which these POA glutamatergic neurons are activated. This could be either in terms of firing frequency, or duration.

A drop in core temperature preceding sleep onset is also observed in humans ( Campbell & Broughton, 1994; Kräuchi, 2007; Landolt *et al*., 1995). Hence, glutamatergic neurons in the medial preoptic area might represent a common circuit that links NREM sleep onset, the core body temperature alterations associated with sleep, and torpor. In keeping with this hypothesis, Takahashi *et al.* ( Takahashi *et al*., 2020) observed disrupted diurnal temperature variation in mice in which POA Qrfp neurotransmission was blocked. It would be very interesting to establish the degree of overlap between the POA glutamatergic neurons that drive NREM sleep and cooling as identified by Harding *et al.* ( Harding *et al*., 2018), and the torpor-inducing neurons identified by Hrvatin *et al*., and Takahashi *et al.*

In order to contribute to torpor induction, POA circuits with a role in thermoregulation and sleep induction within the POA would need to also receive information regarding the nutritional status of the animal. Such information might come from circulating leptin, receptors for which are indeed found on hypothermia-inducing glutamatergic neurons in the POA ( Yu *et al*., 2016).

The dorsomedial hypothalamus is also well-placed to contribute to torpor, and a projection from the POA to DMH is capable of driving reduced body temperature ( Takahashi *et al*., 2020; Tan *et al*., 2016; Zhao *et al*., 2017). As well as playing a role in thermoregulation ( Jeong *et al*., 2015; Liedtke, 2017; Zhao *et al*., 2017), the DMH also adjusts circadian rhythms based on the timing of food availability ( Gooley *et al*., 2006). One might speculate that the dorsomedial hypothalamus integrates information about the availability and timing of food in order to optimise the timing of torpor, which is known to be under circadian control but can be adjusted according to food availability ( van der Vinne *et al*., 2018).

**Figure 3.**
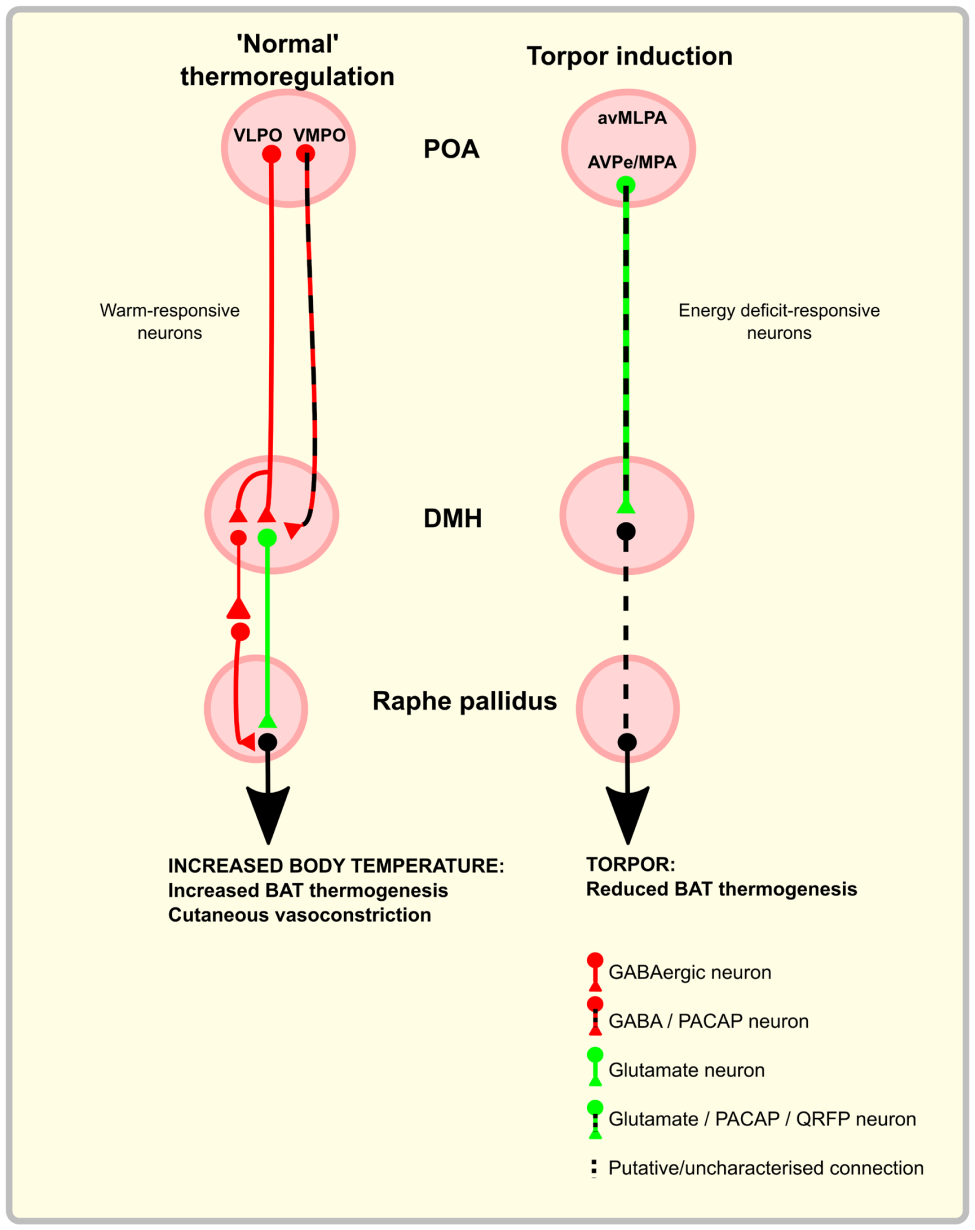
Schematic comparing thermoregulation with torpor induction pathways. The current thermoregulatory model (left) proposes predominantly GABAergic projections from POA to DMH. These GABAergic projections are activated by skin, viscera, or CNS warming and pyrogens. DMH contains both glutamatergic and GABAergic neurons, the activation of which causes increased BAT thermogenesis, vasoconstriction, and increased core temperature. The location of the second GABAergic neuron in the relay from DMH to raphe pallidus has not been established. The emerging model for torpor induction (right) suggests glutamatergic / PACAP / QRFP neurons project from POA to DMH. Similar to the effects of activating the GABAergic POA to DMH, activating this excitatory POA to DMH pathway reduces body temperature, and in this case, induces torpor. The nature of the DMH neurons that are activated by the excitatory POA to DMH projection remains unknown. Abbreviations: GABA, gamma-aminobutyric acid; POA, preoptic area; DMH, dorsomedial hypothalamic area; CNS, central nervous system; BAT, brown adipose tissue; PACAP, Pituitary adenylate-cyclase-activating polypeptide; QRFP, pyroglutamylated RFamide peptide (QRFP).

This proposal that POA to DMH projections may be involved in torpor induction differs from the more established model of thermoregulation in several interesting ways ( Saper & Machado, 2020). Current understanding of homeostatic thermoregulation proposes that a predominantly GABAergic warm-sensing projection from the POA synapses on both GABAergic and glutamatergic neurons in the DMH ( Tan *et al*., 2016; Zhao *et al*., 2017). Activation of either the glutamatergic or the GABAergic neurons in the DMH drives thermogenesis ( Zhao *et al*., 2017). Hence, core temperature is determined by the balance between, on the one hand, the activity of DMH glutamatergic and GABAergic neurons, both of which drive thermogenesis, and on the other hand, the inhibitory input from the POA GABAergic neurons, which suppresses this thermogenic activity in the DMH ( Figure 1). In contrast, the POA to DMH projection that has been implicated in torpor appears to involve predominantly glutamatergic neurons, which express PACAP and / or Qrfp ( Hrvatin *et al*., 2020; Takahashi *et al*., 2020) ( Figure 3). Activation of this presumably excitatory POA to DMH pathway may contribute to the hypothermia associated with torpor ( Takahashi *et al*., 2020). The nature of the DMH neurons targeted by this projection is unknown, but they appear to have antagonistic effects to the populations of glutamatergic and GABAergic DMH neurons implicated in homeostatic thermoregulation ( Zhao *et al*., 2017). That is to say, previously identified DMH neurons - be they glutamatergic or GABAergic – are thought to drive thermogenesis ( Zhao *et al*., 2017), whereas the population targeted by the excitatory PACAP / Qrfp projection from the POA appear to induce hypothermia and torpor. One possibility is that these hypothermia-inducing DMH neurons are cholinergic ( Jeong *et al*., 2015). 

## Conclusions

The field is gaining momentum as new techniques, particularly in the field of systems neuroscience, allow manipulation of the activity of specific hypothalamic neurons. There are, however, several outstanding questions. The lack of an agreed definition of torpor is problematic for the field. While a simple threshold of core temperature has been used, this approach fails to account for the circadian fluctuations in body temperature and fails to distinguish torpor from pathological hypothermia induced by disturbances to normal thermoregulation. This approach also ignores the many additional characteristics of torpor such as active cardiorespiratory suppression, suppression of mitochondrial oxygen consumption, and behavioural changes.

We propose that there are two key features of torpor, which are relatively easily measured, and which might serve as a pragmatic proxy for identifying torpor: reduced body temperature with ongoing active thermoregulation such that a minimum (albeit adjusted) core temperature is defended; and controlled cardiovascular depression such that a characteristic heart rate versus body temperature hysteresis curve is observed ( Vicent *et al*., 2017b).

Another outstanding question is whether daily torpor and seasonal hibernation are manifestations of the same process. While some groups argue that these are mechanistically distinct ( Sunagawa & Takahashi, 2016), this is not widely agreed. It is also unclear whether there exists a torpor ‘master switch’ capable of triggering the full spectrum of associated physiological adaptations, or whether several parallel regions detect the signal for torpor entry and independently trigger components of the response. While current evidence converges on the POA playing a key role ( Hrvatin *et al*., 2020; Takahashi *et al*., 2020; Zhang *et al*., 2020), these studies have tended to focus on body temperature as a proxy for the more complex adaptations associated with natural torpor. If the POA does indeed contain the torpor master switch, whether a projection from here to the DMH ( Takahashi *et al*., 2020) is sufficient for inducing all aspects of torpor is also unknown, and represents an important question.

Finally, although we are beginning to identify some of the neuronal circuits responsible for torpor entry, the mechanism that transduces the signal from deplete energy stores to the POA and wider central nervous system remains unknown. Possible candidates include leptin, ghrelin, NPY, and adenosine, but a necessary and sufficient role for any of these signals has not been demonstrated. Furthermore, we do not yet know whether a single mechanism induces and maintains torpor, with arousal occurring when that mechanism ceases to be engaged, or on the other hand, whether separate processes govern these different stages of torpor.

Better understanding of the characteristics or torpor and the neural and endocrine mechanisms that control it may pave the way to mimicking this intriguing physiological state in humans, with potentially profound applications in medicine ( Aslami & Juffermans, 2010; Lee, 2008; Stanzani *et al*., 2020) and long distance space travel ( Cerri *et al*., 2021b) .

## Data availability

No data are associated with this article.
