# Supplementary material for: Turn it off and on again: characteristics and control of torpor
Source: Wellcome Open Res. 2022 Mar 29;6:313. Originally published 2021 Nov 17. [Version 2] doi: 10.12688/wellcomeopenres.17379.2 (PMC8764563; doi:10.12688/wellcomeopenres.17379.2)
Supplement: Peer review history

# Peer review: Reviewer response for version 2

**DOI:** 10.21956/wellcomeopenres.19655.r49540

**Published:** 2022-04-07

**Authors:** Christopher Madden

**Affiliations:** 1 Department of Neurological Surgery, Oregon Health & Science University, Portland, OR, USA

All previous comments have been adequately addressed.

Is the review written in accessible language?

Yes

Are all factual statements correct and adequately supported by citations?

Partly

Are the conclusions drawn appropriate in the context of the current research literature?

Partly

Is the topic of the review discussed comprehensively in the context of the current literature?

Partly

Reviewer Expertise:

Thermoregulation, autonomic function, brown adipose tissue

I confirm that I have read this submission and believe that I have an appropriate level of expertise to confirm that it is of an acceptable scientific standard.

# Peer review: Reviewer response for version 1

**DOI:** 10.21956/wellcomeopenres.19215.r47146

**Published:** 2022-01-17

**Authors:** David Bechtold

**Affiliations:** 1 Centre for Biological Timing, Faculty of Biology, Medicine and Health, University of Manchester, Manchester, UK

Ambler and colleagues provide an extensive review on daily torpor - a dramatic state of hypothermia and hypometabolism employed by a range of animals in response to challenging environmental conditions. Despite recent advances, there remain fundamental gaps in our understanding about how torpor is regulated, including the physiological conditions and neural circuits that may control entry into and emergence from the torpid state. This review draws together a range of studies on seasonal and fast-induced torpor to consider what putative processes may be involved. The review provides a valuable resource for researchers covering many aspects of torpor regulation (from defining basic characteristic of the torpor state, discussing physiological and/or hormonal factors which may contribute to torpor biology, through to neural pathways that have recently been linked to the expression of torpor in mice).

 As with any review, there is a more that could have been included. For example, inclusion of some earlier torpor research detailing the thermodynamic and physiological adaptions observed during torpor entry may help to inform on what processes are primary drivers vs secondary consequences of the hypometabolic state. Also more direct comparisons throughout the review between natural seasonal torpor (such as observed in hamsters) and fast-induced torpor in mice would be valuable. Nevertheless, the review is impressive and, overall, achieves its purpose of summarising current understanding of how torpor may be regulated.

 I have some comments and suggestions for the authors’ consideration -

1. The authors should take much more care in their early discussions about what defines torpor and its characteristics. In the first line of the introduction, the authors state that torpor is observed “when the metabolic demands of maintaining homeostasis cannot be met”. This is not accurate as stated. In the first paragraph of the discussion ‘physiological characteristics of torpor’, there is similar generalisation which will not be helpful for readers who are not well acquainted with torpor biology. As the authors subsequently point out and discuss, torpor is much more complex process, which is highly regulated and timed. The intro and this first discussion paragraph should contain a more nuanced description of why/when animals may engage torpor as a means of energy conservation. 
2. Similar to point 1, the authors should be careful about mixing observations from different models and generalising findings. For example, “duration of torpor is inversely proportional to the weight of the mouse”, “torpor typically persists for between four to six hours…” “bouts may last up to 12 hours or more…” “low birth weight predicts higher torpor tendency…” “…evidence that torpor is engaged when…fasting decreases body weight below 20g”. The relationship between overall body weight, body weight loss, fat mass etc are all important factors involved in manifestation of torpor. However, these independent observations cannot necessarily be generalised (e.g. CD1 mice have a relatively large body size compared to C57B6 mice, but more readily exhibit torpor upon fasting). Similarly, robust daily torpor can often be observed during chronic food entrainment studies in mice, where steady body weight can be maintained. Clearly, not all studies and findings across different models can be integrated, but where possible the authors could try to draw together important conceptual considerations. For example, in the case of body weight loss – a key aspect is likely to be the development of a ‘torpor permissive’ metabolic state, rather than achieving any absolute threshold of body weight or weight loss. 
3. An important issue discussed by the authors is how torpor can be (or should be) defined in experimental studies. In the final section of the review, the author provide some potential criteria – “reduced body temperature with ongoing thermoregulation such that a minimum (albeit adjusted) core temperature is defended; and controlled cardiovascular depression…”. In my opinion, this first criteria is an important one. Torpor is a highly regulated process, and a relatively stable low body temperature is a clear feature of a ‘full’ torpor bout. This may be an important point of distinction between simple fasting-induced reductions in body temperature and metabolic rate versus entry into a state of torpor. However, why do the authors not then use this criteria when assessing and comparing studies included in their review. For example, when discussing the potential role of leptin in torpor, the authors compare studies of Gavrilova *et al *1999 and Bechtold *et al* 2012. However, they are trying to draw conclusions from wild-type mice which do not appear to enter torpor in either study (as expected from normal mice under short term fasting, they show reduced BT and metabolism, but not torpor). Similar consideration should be applied to studies discussed in the context of NPY signalling, adenosine signalling, etc. With regard to the second criteria (related to the temporal dynamics of heart rate and body temperature change), this is a valuable suggestion, although there are additional considerations required. This relationship needs to be studied in more detail and under additional experimental conditions (species, ambient temperature, fasting vs seasonal torpor, etc). In addition, many laboratories may not have the facilities to collect continuous heart rate in undisturbed animals (e.g. via radio telemetry).
4. The review includes a number of sections which do not directly discuss torpor. For example, pages 5-11 are principally summarising aspects of general physiology. While agreeing with the authors’ comments about a need to understand basic neural and hormonal control mechanisms (e.g. involved in feeding and/or thermoregulation) in order to evaluate how torpor may be controlled, in its current form these relatively long background sections detract somewhat from the review. These sections provide some context, but they do not really provide a level of depth required to add significant insight. Shortening these sections (with references to point the reader to recent in-depth reviews on the subjects) and integrating essential information more directly into discussions on torpor would be beneficial.
5. The most valuable parts of the review are the ‘in summary’ paragraphs which conclude each section. These offer good insight and draw findings together. It would be valuable to give these even more emphasis, while perhaps cutting down on some of the preceding information. This is especially true of discussions around the concept of a ‘torpor-permissive’ physiological states. Accumulated evidence suggests that employment of torpor requires a permissive metabolic state and optimal circadian time window, within which neural drivers of the torpid state can act. This concept is highlighted at a few points during the review, but this could be discussed in more depth and earlier in the review.
6. Fasting induced torpor in mice raises an intriguing issue – the animals undergo a major (and relatively rapid) switch in behaviour and physiology away from foraging into profound energy conservation. The authors may want to more directly discuss this physiological ‘switch’ and how the neural circuitry discussed may be involved.
7. There are some good studies (e.g. by Vladyslav Vyazovskiy and colleagues) exploring the relationship between torpor and sleep in both seasonal (hamster) and mouse studies. These could be included for increased insight. It seems clear that torpor is not a modified state of sleep, but as the authors suggest in the review, there is an important interaction between these processes. For example, could sleep be a necessary transition state into torpor?
8. Figure 2 – the bubble at the top related to “sensory cues indicating food” is perhaps too limited, or need further explanation. Numerous contextual and environmental factors will contribute to foraging drive and/or expectation of feeding. Further, there are a large number of nutrient, hormonal and neural inputs to the hypothalamus and brainstem which influence metabolic control centres in the brain. The authors may want to add more inputs to this circuit. The figure title should also specify ‘hypothalamic circuits’ as other areas are not included. Including the VMH would be appropriate, as circuits for rhythmic regulation of thermogenesis and feeding control have been demonstrated.
9. Figure 3 could include some indication that circadian input and metabolic state can enhance or oppose the ‘torpor-inducing’ pathway.
10. The authors may consider discussing findings related to Gpr50 and torpor. Mice lacking this receptor do not exhibit any major metabolic or physiological phenotype under normal fed conditions, but readily employ deep torpor when fasted. Moreover, the expression of torpor in response to fasting is extremely consistent and shows strong circadian timing. Beyond the specific function of Gpr50, this suggests that torpor may be a ‘default’ response of mice to fasting, but one that is opposed/suppressed under sub-optimal internal or external conditions. It is also noteworthy that the receptor is highly expressed in the DMH and tanycytes of the 3 ^rd^ ventricle – areas which play a major role in seasonal adaptation and torpor.
11. Discussion of circadian rhythmicity in ghrelin and leptin production could be included (could this contribute switch from foraging to conservation, and the associated timing of torpor)?
12. In discussion of NPY administration causing a decrease in food intake (pg 13; in reference to Paul *et al* 2005), the authors should acknowledge that in these studies food intake was assessed 24 hour after administration, and therefore may reflect secondary effects (rather than direct action of NPY on orexigenic circuits).
13. Discussion of A-Zip/F-1 mice should be included with caution. This is a model of extreme lipodystrophy and many animals die during fasting.
14. It seems odd to have the reference appearing at the end of a series of sentences discussing a particular studies (rather than at the first mention). Unless this is standard for the journal, it should be addressed to ensure that the source and context of the information is clear (e.g. final paragraph of page 12, the source of the Siberian hamster studies are not clear until the following page).
15. References should not include an undergraduate thesis (Vincent *et al* 2017a). Also, formatting across citations in the reference list is not consistent. Some include contact information for the corresponding authors and some do not.

Is the review written in accessible language?

Yes

Are all factual statements correct and adequately supported by citations?

Partly

Are the conclusions drawn appropriate in the context of the current research literature?

Yes

Is the topic of the review discussed comprehensively in the context of the current literature?

Partly

Reviewer Expertise:

Animal physiology, circadian biology, energy metabolism, neural controls of energy balance.

I confirm that I have read this submission and believe that I have an appropriate level of expertise to confirm that it is of an acceptable scientific standard, however I have significant reservations, as outlined above.

# Response

**Published:** 2022-01-24

**Authors:** Mike Ambler

**Affiliations:** School of Physiology, Pharmacology and Neuroscience, University of Bristol, UK

We thank the reviewer for their comments, while the manuscript has now reached the point of publication, we will nevertheless incorporate the suggestions and attempt to address the comments in an updated manuscript.

# Response

**Published:** 2022-03-09

**Authors:** Mike Ambler

**Affiliations:** School of Physiology, Pharmacology and Neuroscience, University of Bristol, UK

Ambler and colleagues provide an extensive review on daily torpor - a dramatic state of hypothermia and hypometabolism employed by a range of animals in response to challenging environmental conditions. Despite recent advances, there remain fundamental gaps in our understanding about how torpor is regulated, including the physiological conditions and neural circuits that may control entry into and emergence from the torpid state. This review draws together a range of studies on seasonal and fast-induced torpor to consider what putative processes may be involved. The review provides a valuable resource for researchers covering many aspects of torpor regulation (from defining basic characteristic of the torpor state, discussing physiological and/or hormonal factors which may contribute to torpor biology, through to neural pathways that have recently been linked to the expression of torpor in mice).

 As with any review, there is a more that could have been included. For example, inclusion of some earlier torpor research detailing the thermodynamic and physiological adaptions observed during torpor entry may help to inform on what processes are primary drivers vs secondary consequences of the hypometabolic state. Also more direct comparisons throughout the review between natural seasonal torpor (such as observed in hamsters) and fast-induced torpor in mice would be valuable. Nevertheless, the review is impressive and, overall, achieves its purpose of summarising current understanding of how torpor may be regulated.

 I have some comments and suggestions for the authors’ consideration -

1. The authors should take much more care in their early discussions about what defines torpor and its characteristics. In the first line of the introduction, the authors state that torpor is observed “when the metabolic demands of maintaining homeostasis cannot be met”. This is not accurate as stated. In the first paragraph of the discussion ‘physiological characteristics of torpor’, there is similar generalisation which will not be helpful for readers who are not well acquainted with torpor biology. As the authors subsequently point out and discuss, torpor is much more complex process, which is highly regulated and timed. The intro and this first discussion paragraph should contain a more nuanced description of why/when animals may engage torpor as a means of energy conservation. 

***We have adjusted the phrasing in these sections taking into account these comments, and removing the generalisation that torpor is engaged in simply in response to food restriction.***

1. Similar to point 1, the authors should be careful about mixing observations from different models and generalising findings. For example, “duration of torpor is inversely proportional to the weight of the mouse”, “torpor typically persists for between four to six hours…” “bouts may last up to 12 hours or more…” “low birth weight predicts higher torpor tendency…” “…evidence that torpor is engaged when…fasting decreases body weight below 20g”. The relationship between overall body weight, body weight loss, fat mass etc are all important factors involved in manifestation of torpor. However, these independent observations cannot necessarily be generalised (e.g. CD1 mice have a relatively large body size compared to C57B6 mice, but more readily exhibit torpor upon fasting). Similarly, robust daily torpor can often be observed during chronic food entrainment studies in mice, where steady body weight can be maintained. Clearly, not all studies and findings across different models can be integrated, but where possible the authors could try to draw together important conceptual considerations. For example, in the case of body weight loss – a key aspect is likely to be the development of a ‘torpor permissive’ metabolic state, rather than achieving any absolute threshold of body weight or weight loss. 

***We have added these nuances to the section discussing the timing of / triggers for torpor.***

1. An important issue discussed by the authors is how torpor can be (or should be) defined in experimental studies. In the final section of the review, the author provide some potential criteria – “reduced body temperature with ongoing thermoregulation such that a minimum (albeit adjusted) core temperature is defended; and controlled cardiovascular depression…”. In my opinion, this first criteria is an important one. Torpor is a highly regulated process, and a relatively stable low body temperature is a clear feature of a ‘full’ torpor bout. This may be an important point of distinction between simple fasting-induced reductions in body temperature and metabolic rate versus entry into a state of torpor. However, why do the authors not then use this criteria when assessing and comparing studies included in their review. For example, when discussing the potential role of leptin in torpor, the authors compare studies of Gavrilova *et al *1999 and Bechtold *et al* 2012. However, they are trying to draw conclusions from wild-type mice which do not appear to enter torpor in either study (as expected from normal mice under short term fasting, they show reduced BT and metabolism, but not torpor).Similar consideration should be applied to studies discussed in the context of NPY signalling, adenosine signalling, etc.

***We have pointed out in the section discussing these studies that in the WT mice what is observed is a reduction in metabolic rate and temperature but probably not torpor.***

 With regard to the second criteria (related to the temporal dynamics of heart rate and body temperature change), this is a valuable suggestion, although there are additional considerations required. This relationship needs to be studied in more detail and under additional experimental conditions (species, ambient temperature, fasting vs seasonal torpor, etc). In addition, many laboratories may not have the facilities to collect continuous heart rate in undisturbed animals (e.g. via radio telemetry).

***We agree that temporal dynamics of heart rate vs temperature during torpor entry vs exit is important, and have added an acknowledgement that measuring heart rate is not always possible. However, we do feel that this feature is of sufficient importance to justify the proposal that it could form part of a criteria for defining torpor.  ***

1. The review includes a number of sections which do not directly discuss torpor. For example, pages 5-11 are principally summarising aspects of general physiology. While agreeing with the authors’ comments about a need to understand basic neural and hormonal control mechanisms (e.g. involved in feeding and/or thermoregulation) in order to evaluate how torpor may be controlled, in its current form these relatively long background sections detract somewhat from the review. These sections provide some context, but they do not really provide a level of depth required to add significant insight. Shortening these sections (with references to point the reader to recent in-depth reviews on the subjects) and integrating essential information more directly into discussions on torpor would be beneficial.

**We have edited down these sections but have kept them as an introduction for those less familiar. We hope that by using specific headings, readers will be able to choose whether or not to concentrate on the sections discussing general physiology.**

1. The most valuable parts of the review are the ‘in summary’ paragraphs which conclude each section. These offer good insight and draw findings together. It would be valuable to give these even more emphasis, while perhaps cutting down on some of the preceding information. This is especially true of discussions around the concept of a ‘torpor-permissive’ physiological states. Accumulated evidence suggests that employment of torpor requires a permissive metabolic state and optimal circadian time window, within which neural drivers of the torpid state can act. This concept is highlighted at a few points during the review, but this could be discussed in more depth and earlier in the review.
2. Fasting induced torpor in mice raises an intriguing issue – the animals undergo a major (and relatively rapid) switch in behaviour and physiology away from foraging into profound energy conservation. The authors may want to more directly discuss this physiological ‘switch’ and how the neural circuitry discussed may be involved.

***We have added reference to this profound switch in the introduction.***

1. There are some good studies (e.g. by Vladyslav Vyazovskiy and colleagues) exploring the relationship between torpor and sleep in both seasonal (hamster) and mouse studies. These could be included for increased insight. It seems clear that torpor is not a modified state of sleep, but as the authors suggest in the review, there is an important interaction between these processes. For example, could sleep be a necessary transition state into torpor?

**We have included reference to these studies from Vlad Vyazovskiy, and discussed the idea that NREM is a transition state.**

1. Figure 2 – the bubble at the top related to “sensory cues indicating food” is perhaps too limited, or need further explanation. Numerous contextual and environmental factors will contribute to foraging drive and/or expectation of feeding. Further, there are a large number of nutrient, hormonal and neural inputs to the hypothalamus and brainstem which influence metabolic control centres in the brain. The authors may want to add more inputs to this circuit. The figure title should also specify ‘hypothalamic circuits’ as other areas are not included. Including the VMH would be appropriate, as circuits for rhythmic regulation of thermogenesis and feeding control have been demonstrated

**We have edited the figure but are reluctant to add too many new inputs without discussing them in the body of the text, and do not wish to over-burden the reader with more general physiology beyond what is already included.   **

1. Figure 3 could include some indication that circadian input and metabolic state can enhance or oppose the ‘torpor-inducing’ pathway.

**We have edited the figure to highlight the sensitivity to metabolic state and circadian time.**

1. The authors may consider discussing findings related to Gpr50 and torpor. Mice lacking this receptor do not exhibit any major metabolic or physiological phenotype under normal fed conditions, but readily employ deep torpor when fasted. Moreover, the expression of torpor in response to fasting is extremely consistent and shows strong circadian timing. Beyond the specific function of Gpr50, this suggests that torpor may be a ‘default’ response of mice to fasting, but one that is opposed/suppressed under sub-optimal internal or external conditions. It is also noteworthy that the receptor is highly expressed in the DMH and tanycytes of the 3 ^rd^ ventricle – areas which play a major role in seasonal adaptation and torpor.

**We have included a discussion of these experiments in the leptin and torpor section.**

1. Discussion of circadian rhythmicity in ghrelin and leptin production could be included (could this contribute switch from foraging to conservation, and the associated timing of torpor)?
2. In discussion of NPY administration causing a decrease in food intake (pg 13; in reference to Paul *et al* 2005), the authors should acknowledge that in these studies food intake was assessed 24 hour after administration, and therefore may reflect secondary effects (rather than direct action of NPY on orexigenic circuits).

**We have added this useful point.**

1. Discussion of A-Zip/F-1 mice should be included with caution. This is a model of extreme lipodystrophy and many animals die during fasting.
2. It seems odd to have the reference appearing at the end of a series of sentences discussing a particular studies (rather than at the first mention). Unless this is standard for the journal, it should be addressed to ensure that the source and context of the information is clear (e.g. final paragraph of page 12, the source of the Siberian hamster studies are not clear until the following page).
3. References should not include an undergraduate thesis (Vincent *et al* 2017a). Also, formatting across citations in the reference list is not consistent. Some include contact information for the corresponding authors and some do not.

**We have corrected this and the point above.**

# Peer review: Reviewer response for version 1

**DOI:** 10.21956/wellcomeopenres.19215.r47150

**Published:** 2021-12-24

**Authors:** Kelly Drew

**Affiliations:** 1 Department of Chemistry and Biochemistry, Institute of Arctic Biology, Center for Transformative Research in Metabolism, University of Alaska Fairbanks, Fairbanks, AK, USA

This is a comprehensive review aimed at introducing readers to torpor and bringing them up to speed on current knowledge of the central processes that coordinate torpor onset. Overall, the manuscript is well written, easy to follow and provides a timely update on the CNS control of torpor. Major concerns stem primarily from an attempt to squeeze all types of torpor into a single box and errors made in describing some of the nuances of hibernation.

**Major concerns that must be addressed to make the article sound are as follows:**

 1. The authors write, “…an assumption is that the torpor during these hibernation periods is mechanistically equivalent to the brief torpor bouts seen in daily hetereotherms such as the mouse. However, this assumption has not been experimentally proven."

- This statement distracts from the otherwise careful consideration of the literature that is reviewed. A preferred approach would be to create a table that compares and contrasts daily torpor, facultative hibernation and obligate hibernation and identify what, if any, attributes these three types of torpor share.

2. The authors discuss hibernation in hamsters as “seasonal” and suggest that hibernation is a response to environmental challenge:

- Syrian hamsters are termed facultative hibernators. The authors should investigate this term, define it for readers and apply the definition in their review. In particular, consider describing facultative and obligate hibernation with reference to gonadal regression; one of many features the authors will find distinguishes hibernation from daily heterothermy.
- Note that obligate hibernators do NOT ‘respond” to environmental challenge. No degree of environmental challenge will induce hibernation in the summer season in an obligate hibernator.
- Alternatively, limit the review to fasting-induced torpor in mice.
- The authors suggest in the abstract that they offer a “critical’ view of evidence for the systems that control torpor entry. However, nowhere in the manuscript is there any critical consideration of qualities that enhance rigor, or mention of which studies followed practices that enhance rigor, such as blinding, inclusion of positive and negative controls, and a priori power analysis. A critical review of studies supporting various models and which models are supported by papers with appropriate control groups and blinding would have a positive impact on the field.

3. The authors suggest that because fasting-induced torpor persists in mice lacking A1R and A3R that adenosine represents a candidate signal for torpor initiation, but once again, must be designating as ‘contributing’ or ‘permissive’ and not a necessary and sufficient master switch. Again, the authors miss an opportunity to “critically” review existing evidence by failing to acknowledge the potential for compensatory mechanisms in the KO strains used by Carlin *et al.* ^1^, and the call for further study using conditional KO experiments.

 4. The authors propose two key features of torpor; 1. Reduced body temperature with active, ongoing thermogenesis to defend a lower Tb, and 2. Characteristic hysteresis of heart rate and core body temperature. Unfortunately, they do not offer any rationale for selecting these two features other than that they are easily measured. I agree that a decrease in metabolic rate, for which heart rate is a proxy during entrance, should precede a decline in body temperature. However, the same signal that induces torpor may not be involved with arousal. While the classic hysteresis may be easy to measure using it to identify torpor, importantly experimentally- induced torpor, assumes that torpor onset and arousal are regulated by the same mechanism. No evidence that I am aware of would suggest this is the case. Indeed, despite the recent focus and progress in identifying circuits involved with the onset of fasting-induced torpor in mice, few studies have focused on mechanisms or circuits involved in arousal. The hysteresis curve illustrates two processes, onset of torpor and arousal from torpor. I would suggest that a combination of metabolic suppression and vasoconstriction would be a better characteristic to distinguish torpor from hypothermia because a warm defense response suppresses thermogenesis and increases vasodilation, while energy conserving torpor suppresses thermogenesis while limiting heat loss through vasoconstriction.

 5. The review is missing mention of thermoregulatory inversion, a model that describes the phenomenon of altered thermoregulation during torpor onset (see Tupone *et al.* 2017 ^2^). The description of this phenomenon could prove to be the characteristic that defines torpor and would likely be of interest to readers.

 6. The section on Sleep and Torpor is missing an alternative interpretation that sleep debt does not accumulate during torpor (for example see Larkin and Heller, 1999 ^3^).

 7. Lacking is any attention to the phenotype of an animal prior to torpor onset. Should pharmacological manipulation of fed mice be expected to replicate effects in fasted mice?

**Other concerns:**

 1. Reference to Fig. 1 (page 2, 2 ^nd^ paragraph) suggests that Fig. 1 should include a phylogenic tree, but instead we see a schematic of the thermal defense circuit.

 2. The authors write, “For a given body temperature, pharmacologically-induced hypothermia generates a less profound bradycardia than that seen in torpor…” - What kind of pharmacologically-induced hypothermia? In what species? Under what conditions?

Is the review written in accessible language?

Yes

Are all factual statements correct and adequately supported by citations?

Partly

Are the conclusions drawn appropriate in the context of the current research literature?

Partly

Is the topic of the review discussed comprehensively in the context of the current literature?

Yes

Reviewer Expertise:

Hibernation, neural control of hibernation

I confirm that I have read this submission and believe that I have an appropriate level of expertise to state that I do not consider it to be of an acceptable scientific standard, for reasons outlined above.

## References

[1] : Hypothermia in mouse is caused by adenosine A1 and A3 receptor agonists and AMP via three distinct mechanisms. *Neuropharmacology* .2017;114: 10.1016/j.neuropharm.2016.11.026 101-113 10.1016/j.neuropharm.2016.11.026 PMC518355227914963

[2] : Thermoregulatory inversion: a novel thermoregulatory paradigm. *American Journal of Physiology-Regulatory, Integrative and Comparative Physiology* .2017;312(5) : 10.1152/ajpregu.00022.2017 R779-R786 10.1152/ajpregu.00022.2017 PMC545156928330964

[3] : Sleep after arousal from hibernation is not homeostatically regulated. *Am J Physiol* .276(2) : 10.1152/ajpregu.1999.276.2.R522 R522-9 10.1152/ajpregu.1999.276.2.R522 9950933

# Response

**Published:** 2022-01-24

**Authors:** Mike Ambler

**Affiliations:** School of Physiology, Pharmacology and Neuroscience, University of Bristol, UK

We thank the reviewer for their comments and will incorporate them into an updated manuscript.

# Response

**Published:** 2022-03-09

**Authors:** Mike Ambler

**Affiliations:** School of Physiology, Pharmacology and Neuroscience, University of Bristol, UK

Kelly Drew

 This is a comprehensive review aimed at introducing readers to torpor and bringing them up to speed on current knowledge of the central processes that coordinate torpor onset. Overall, the manuscript is well written, easy to follow and provides a timely update on the CNS control of torpor. Major concerns stem primarily from an attempt to squeeze all types of torpor into a single box and errors made in describing some of the nuances of hibernation.

Major concerns that must be addressed to make the article sound are as follows:

 1. The authors write, “…an assumption is that the torpor during these hibernation periods is mechanistically equivalent to the brief torpor bouts seen in daily hetereotherms such as the mouse. However, this assumption has not been experimentally proven."

- This statement distracts from the otherwise careful consideration of the literature that is reviewed. A preferred approach would be to create a table that compares and contrasts daily torpor, facultative hibernation and obligate hibernation and identify what, if any, attributes these three types of torpor share.

**We have revised the manuscript to highlight some of the differences between daily torpor and hibernation.**

 2. The authors discuss hibernation in hamsters as “seasonal” and suggest that hibernation is a response to environmental challenge:

- Syrian hamsters are termed facultative hibernators. The authors should investigate this term, define it for readers and apply the definition in their review. In particular, consider describing facultative and obligate hibernation with reference to gonadal regression; one of many features the authors will find distinguishes hibernation from daily heterothermy.
- Note that obligate hibernators do NOT ‘respond” to environmental challenge. No degree of environmental challenge will induce hibernation in the summer season in an obligate hibernator.

**We have added some discussion of daily torpor vs hibernation (including a table comparing these phenomena).**
* *

- Alternatively, limit the review to fasting-induced torpor in mice.
- The authors suggest in the abstract that they offer a “critical’ view of evidence for the systems that control torpor entry. However, nowhere in the manuscript is there any critical consideration of qualities that enhance rigor, or mention of which studies followed practices that enhance rigor, such as blinding, inclusion of positive and negative controls, and a priori power analysis. A critical review of studies supporting various models and which models are supported by papers with appropriate control groups and blinding would have a positive impact on the field.

**We have removed this comment.**

 3. The authors suggest that because fasting-induced torpor persists in mice lacking A1R and A3R that adenosine represents a candidate signal for torpor initiation, but once again, must be designating as ‘contributing’ or ‘permissive’ and not a necessary and sufficient master switch. Again, the authors miss an opportunity to “critically” review existing evidence by failing to acknowledge the potential for compensatory mechanisms in the KO strains used by Carlin *et al.* ^1^ , and the call for further study using conditional KO experiments.

*We have acknowledged this point regarding KO strains.*

 4. The authors propose two key features of torpor; 1. Reduced body temperature with active, ongoing thermogenesis to defend a lower Tb, and 2. Characteristic hysteresis of heart rate and core body temperature. Unfortunately, they do not offer any rationale for selecting these two features other than that they are easily measured.

**Our rationale was wanting to identify characteristics of torpor that were relatively easily measured experimentally.**

 I agree that a decrease in metabolic rate, for which heart rate is a proxy during entrance, should precede a decline in body temperature.

*In our opinion, a reduced heart rate is more than a proxy for reduced metabolic rate but represents a fundamental component of torpor.*

 However, the same signal that induces torpor may not be involved with arousal. While the classic hysteresis may be easy to measure using it to identify torpor, importantly experimentally- induced torpor, assumes that torpor onset and arousal are regulated by the same mechanism. No evidence that I am aware of would suggest this is the case. Indeed, despite the recent focus and progress in identifying circuits involved with the onset of fasting-induced torpor in mice, few studies have focused on mechanisms or circuits involved in arousal. The hysteresis curve illustrates two processes, onset of torpor and arousal from torpor.

**We agree that torpor arousal may be under the control of distinct neural circuits. Our proposed definition is not making any comment on the mechanisms of control, rather we are trying to identify characteristic features of torpor (entry and arousal) against which experimental models might be compared. The data that suggest a characteristic hysteresis curve of heart rate versus temperature is derived from mice entering natural torpor compared to pharmacologically or physically-induced hypothermia (Vicent, Borre and Swoap, 2017), hence these data reflect the natural processes of torpor induction and arousal.**

 I would suggest that a combination of metabolic suppression and vasoconstriction would be a better characteristic to distinguish torpor from hypothermia because a warm defense response suppresses thermogenesis and increases vasodilation, while energy conserving torpor suppresses thermogenesis while limiting heat loss through vasoconstriction.

**We agree that vasoconstriction is probably a crucial component of natural torpor (Swoap and Gutilla, 2009), and that crucially some experimental models of chemo- or optogenetic torpor induction appear to demonstrate vasodilatation (at least in the early phase), which may not represent natural torpor induction (Takahashi et al., 2020; Zhang et al., 2020). However, demonstrating vasoconstriction is not easy experimentally, and for this reason we did not include initially. We have now added this component to the manuscript.**

 5. The review is missing mention of thermoregulatory inversion, a model that describes the phenomenon of altered thermoregulation during torpor onset (see Tupone *et al.* 2017 ^2^ ). The description of this phenomenon could prove to be the characteristic that defines torpor and would likely be of interest to readers.

**In the interest of keeping to manuscript to a reasonable length, we did not include this. We do not necessarily feel that thermoregulatory inversion represents a fundamental characteristic of torpor. During torpor thermoregulation is maintained albeit with a lower resulting body temperature: torpid mice will defend a minimum temperature, which is not predicted by thermoregulatory inversion model.**

 6. The section on Sleep and Torpor is missing an alternative interpretation that sleep debt does not accumulate during torpor (for example see Larkin and Heller, 1999 ^3^ ).

**We have included a discussion of this paper.**

 7. Lacking is any attention to the phenotype of an animal prior to torpor onset. Should pharmacological manipulation of fed mice be expected to replicate effects in fasted mice?

**This is an interesting question, we have discussed the triggers for torpor, and made some comparison between daily torpor and hibernation. We have a separate paper in press that explores some of the question regarding interventions in fed vs calorie-restricted mice (preprint https://www.biorxiv.org/content/10.1101/2021.09.05.458994v1).**

Other concerns:

 1. Reference to Fig. 1 (page 2, 2 ^nd^ paragraph) suggests that Fig. 1 should include a phylogenic tree, but instead we see a schematic of the thermal defense circuit.

***Thank you, we have corrected this error.***

 2. The authors write, “For a given body temperature, pharmacologically-induced hypothermia generates a less profound bradycardia than that seen in torpor…” - What kind of pharmacologically-induced hypothermia? In what species? Under what conditions?

***In mice given CHA – we have clarified in the manuscript.***

# Peer review: Reviewer response for version 1

**DOI:** 10.21956/wellcomeopenres.19215.r47148

**Published:** 2021-12-21

**Authors:** Christopher Madden

**Affiliations:** 1 Department of Neurological Surgery, Oregon Health & Science University, Portland, OR, USA

This manuscript is a well-written, timely, and extensive (although not comprehensive) review of torpor physiology. One major assumption stated in the introduction is that “the torpor during these hibernation periods is mechanistically equivalent to the brief torpor bouts seen in daily heterotherms such as the mouse”, there are data throughout the manuscript that may contradict this assumption (as acknowledged by the authors). Please see the work of Geiser *et al *for additional discussions of this issue (Ann rev physiol 2004 ^1^; Phys Zoology 1995 ^2^). At a minimum this statement that the mechanistics are “equivalent” should be tempered, if not excluded altogether. Below are additional suggestions:

1.  Figure 1. There are data to suggest that many of the BDNF/PACAP neurons express Vglut2 and may be glutamatergic. This possibility should at least be acknowledged.
2. Page 4, first paragraph of the Cardiovascular function during torpor section. The statement is made that slower heart rates during entry into torpor vs arousal from torpor “indicates a dominance of parasympathetic …during entry, followed by sympathetic…during arousal” . Please provide a reference showing parasympathetic involvement during entry, as a lower heart rate could simply be a withdrawal of sympathetic activity. Or at a minimum, use “suggests” instead of “indicates”.
3.  Page 5. The idea of moving away from a set-point model to one of multiple independent mechanisms each with their own thresholds was largely initiated by Romanovsky, it would be appropriate to include a citation to his influential work.
4. Page 5. While relatively little is known about the thermosensitive inputs arriving to the CNS via vagal and splanchnic afferents, there is a more detail description of this issue in my review, Madden & Morrison, (Neurosci Lett 2019) ^3^.
5. Page 6. TRPM2 contributes to temperature sensing in POA neurons. However, genetic deletion of TRPM2 does not affect normal body temperature, therefore it is not necessary for normal euthermia, possibly suggesting that other temperature sensing mechanisms contribute. In addition, TRPM2 has a very broad expression pattern and likely has additional roles beyond temperature sensing. Perhaps these points are beyond the scope of this manuscript, but as this section currently reads, it could be misleading, leading to the possible misinterpretation the TRPM2 are the only mediator of central thermosensing.
6. Page 6 second column first section. “Opto-activation of the warm-sensing GABAergic BDNF and PACAP-expressing cell bodies”. Care should be taken when referring to “warm- sensing” vs. warm-responsive neurons. The BDNF/PACAP cells are responsive to thermal inputs but are not directly warm sensitive (see Tan *et al* 2016 ^4^ “In contrast, we found no evidence that VMPOBDNF/PACAP neurons were more intrinsically thermosensitive than neighbouring cells when we performed whole-cell patch clamp recordings from acute brain slices (Figures S2L and S2M).”)
7. The short-hand “DMH-Raphe-BAT” is potentially confusing since there are synapses between the Raphe and BAT.
8. Page 6 last section. The concept of a maintenance of body weight is perhaps inappropriate. How would a biological system be configured to regulate body weight? It seems likely that the regulated variable would be some other correlate of body weight such as fat mass. Furthermore, the concept that calorie excess increases body temperature is controversial and complicated, and may depend on acute vs. chronic conditions. For example, rats maintained chronically on a high fat diet have reduced sympathetic outflow to BAT (Levin *et al *AJP, 1985 ^5^; Sakaguchi *et al* Physiol Behav, 1989 ^6^; Madden & Morrison AJP, 2016 ^7^ (my paper)).
9. Figure 2. The suggestion that the DMH contributes to energy expenditure and the PVH contributes to food intake is oversimplified. Clearly the paraventricular hypothalamus also influences energy expenditure (BAT) (see, Billington *et al*, 1994 ^8^; Madden & Morrison, 2009 ^9^ (my paper); Nakamura, 2017 ^10^).
10. Page 8. “Selective reintroduction of melanocortin 4 receptors in the PVH normalizes the excess food intake but not the suppressed energy expenditure”. The selective reintroduction of MC4R in the cited work would be in the cell bodies in PVH not in afferent terminals to the PVH so the statement the MC4R is normalized in the PVH is an overinterpretation.
11. Page 10. Again the concept that the Arc to PVH projection is relevant to feeding and that the DMH projection modulates EE is overly simplistic. See the work of Shi *et al *J Neuroendo 2021 ^11^; Billington ^8^; Nakamura ^10^.
12. Page 12, numbered point 2. Beta3 activation would not only activate WAT to suppress leptin but would also activate BAT to generate thermogenesis. 
13. The issue of the role of leptin in torpor is of high importance. Here again it calls into question the assertion that torpor in daily heterotherms and longer duration hibernation bouts are equivalent. Hibernation requires preparatory fattening which would result in elevated leptin levels, on the other hand daily torpor bouts appear to be blocked by leptin administration. 
14. Page 14. Please provide a reference for the statements regarding the incidence of asystoles in torpor and following CHA.
15. The role of endogenous opioids in thermoregulation might benefit from a discussion of the role of kappa opioid receptors in high fat diet induced impairment of BAT activation. (see Conceicao *et al*, eNeuro, 2021 ^12^ (my paper)) and the parallels between animals maintained on a high fat diet and hibernation (see my paper, Madden, Temperature, 2017 ^13^).
16. The suggestion that hypothermia-inducing DMH neurons are cholinergic is insightful and interesting. Nonetheless, the DMH neurons that express fos at torpor initiation are not cholinergic (Hitrec, 2019 ^14^).

Is the review written in accessible language?

Yes

Are all factual statements correct and adequately supported by citations?

Partly

Are the conclusions drawn appropriate in the context of the current research literature?

Partly

Is the topic of the review discussed comprehensively in the context of the current literature?

Partly

Reviewer Expertise:

Thermoregulation, autonomic function, brown adipose tissue

I confirm that I have read this submission and believe that I have an appropriate level of expertise to confirm that it is of an acceptable scientific standard, however I have significant reservations, as outlined above.

## References

[1] : Metabolic rate and body temperature reduction during hibernation and daily torpor. *Annu Rev Physiol* .2004;66: 10.1146/annurev.physiol.66.032102.115105 239-74 10.1146/annurev.physiol.66.032102.115105 14977403

[2] : Hibernation versus Daily Torpor in Mammals and Birds: Physiological Variables and Classification of Torpor Patterns. *Physiological Zoology* .1995;68(6) : 10.1086/physzool.68.6.30163788 935-966 10.1086/physzool.68.6.30163788

[3] : Central nervous system circuits that control body temperature. *Neuroscience Letters* .2019;696: 10.1016/j.neulet.2018.11.027 225-232 10.1016/j.neulet.2018.11.027 PMC639769230586638

[4] : Warm-Sensitive Neurons that Control Body Temperature. *Cell* .2016;167(1) : 10.1016/j.cell.2016.08.028 47-59.e15 10.1016/j.cell.2016.08.028 27616062PMC5062957

[5] : Brown adipose and metabolic features of chronic diet-induced obesity. *Am J Physiol* .1985;248(6 Pt 2) : 10.1152/ajpregu.1985.248.6.R717 R717-23 10.1152/ajpregu.1985.248.6.R717 3890563

[6] : Effect of a high-fat diet on firing rate of sympathetic nerves innervating brown adipose tissue in anesthetized rats. *Physiology & Behavior* .1989;45(6) : 10.1016/0031-9384(89)90106-6 1177-1182 10.1016/0031-9384(89)90106-6 2813541

[7] : A high-fat diet impairs cooling-evoked brown adipose tissue activation via a vagal afferent mechanism. *American Journal of Physiology-Endocrinology and Metabolism* .2016;311(2) : 10.1152/ajpendo.00081.2016 E287-E292 10.1152/ajpendo.00081.2016 PMC500596627354235

[8] : Neuropeptide Y in hypothalamic paraventricular nucleus: a center coordinating energy metabolism. *Am J Physiol* .1994;266(6 Pt 2) : 10.1152/ajpregu.1994.266.6.R1765 R1765-70 10.1152/ajpregu.1994.266.6.R1765 8024026

[9] : Neurons in the paraventricular nucleus of the hypothalamus inhibit sympathetic outflow to brown adipose tissue. *Am J Physiol Regul Integr Comp Physiol* .2009;296(3) : 10.1152/ajpregu.91007.2008 R831-43 10.1152/ajpregu.91007.2008 19129373PMC2854003

[10] : Medullary Reticular Neurons Mediate Neuropeptide Y-Induced Metabolic Inhibition and Mastication. *Cell Metabolism* .2017;25(2) : 10.1016/j.cmet.2016.12.002 322-334 10.1016/j.cmet.2016.12.002 PMC529902828065829

[11] : Neuropeptide Y suppresses thermogenic and cardiovascular sympathetic nerve activity via Y1 receptors in the paraventricular nucleus and dorsomedial hypothalamus. *J Neuroendocrinol* .33(8) : 10.1111/jne.13006 e13006 10.1111/jne.13006 34235800PMC8653878

[12] : Activation of Transient Receptor Potential Vanilloid 1 Channels in the Nucleus of the Solitary Tract and Activation of Dynorphin Input to the Median Preoptic Nucleus Contribute to Impaired BAT Thermogenesis in Diet-Induced Obesity. *eNeuro* .8(2) : 10.1523/ENEURO.0048-21.2021 10.1523/ENEURO.0048-21.2021 33707202PMC8174036

[13] : Brown fat in obesity: Uncoupling protein-1 versus thermogenic activity. *Temperature (Austin)* .2017;4(2) : 10.1080/23328940.2017.1317380 126-127 10.1080/23328940.2017.1317380 28680928PMC5489016

[14] : Neural control of fasting-induced torpor in mice. *Scientific Reports* .2019;9(1) : 10.1038/s41598-019-51841-2 10.1038/s41598-019-51841-2 31664081PMC6820542

# Response

**Published:** 2022-01-24

**Authors:** Mike Ambler

**Affiliations:** School of Physiology, Pharmacology and Neuroscience, University of Bristol, UK

We thank the reviewer for their comments and suggestions. We will update the manuscript to incorporate these points.

# Response

**Published:** 2022-03-09

**Authors:** Mike Ambler

**Affiliations:** School of Physiology, Pharmacology and Neuroscience, University of Bristol, UK

Christopher Madden:

 This manuscript is a well-written, timely, and extensive (although not comprehensive) review of torpor physiology. One major assumption stated in the introduction is that “the torpor during these hibernation periods is mechanistically equivalent to the brief torpor bouts seen in daily heterotherms such as the mouse”, there are data throughout the manuscript that may contradict this assumption (as acknowledged by the authors). Please see the work of Geiser *et al *for additional discussions of this issue (Ann rev physiol 2004 ^1^ ; Phys Zoology 1995 ^2^ ). At a minimum this statement that the mechanistics are “equivalent” should be tempered, if not excluded altogether.

**We have revised how we discuss these data and acknowledged some fundamental differences between torpor and hibernation particularly with respect to the state of calorie deficit vs excess prior to imitation.**

 Below are additional suggestions:

1.  Figure 1. There are data to suggest that many of the BDNF/PACAP neurons express Vglut2 and may be glutamatergic. This possibility should at least be acknowledged.

**We have updated both the figure and the text to discuss this.**

1. Page 4, first paragraph of the Cardiovascular function during torpor section. The statement is made that slower heart rates during entry into torpor vs arousal from torpor “indicates a dominance of parasympathetic …during entry, followed by sympathetic…during arousal” . Please provide a reference showing parasympathetic involvement during entry, as a lower heart rate could simply be a withdrawal of sympathetic activity. Or at a minimum, use “suggests” instead of “indicates”.

**We have provided references for the role of the parasympathetic nervous system in torpor and also adjusted the language.**

1.  Page 5. The idea of moving away from a set-point model to one of multiple independent mechanisms each with their own thresholds was largely initiated by Romanovsky, it would be appropriate to include a citation to his influential work.

**We have included a reference to this work and adjusted the wording of the context.**

1. Page 5. While relatively little is known about the thermosensitive inputs arriving to the CNS via vagal and splanchnic afferents, there is a more detail description of this issue in my review, Madden & Morrison, (Neurosci Lett 2019) ^3^ .

**Thank you we have added this useful reference.**

1. Page 6. TRPM2 contributes to temperature sensing in POA neurons. However, genetic deletion of TRPM2 does not affect normal body temperature, therefore it is not necessary for normal euthermia, possibly suggesting that other temperature sensing mechanisms contribute. In addition, TRPM2 has a very broad expression pattern and likely has additional roles beyond temperature sensing. Perhaps these points are beyond the scope of this manuscript, but as this section currently reads, it could be misleading, leading to the possible misinterpretation the TRPM2 are the only mediator of central thermosensing.

**We have updated the manuscript to acknowledge that TRPM2 is only part of a system that detects local brain temperature. **

1. Page 6 second column first section. “Opto-activation of the warm-sensing GABAergic BDNF and PACAP-expressing cell bodies”. Care should be taken when referring to “warm- sensing” vs. warm-responsive neurons. The BDNF/PACAP cells are responsive to thermal inputs but are not directly warm sensitive (see Tan *et al* 2016 ^4^ “In contrast, we found no evidence that VMPOBDNF/PACAP neurons were more intrinsically thermosensitive than neighbouring cells when we performed whole-cell patch clamp recordings from acute brain slices (Figures S2L and S2M).”)

**Thank you we have clarified this section.**

1. The short-hand “DMH-Raphe-BAT” is potentially confusing since there are synapses between the Raphe and BAT.

**We have clarified this section.**
* *

1. Page 6 last section. The concept of a maintenance of body weight is perhaps inappropriate. How would a biological system be configured to regulate body weight? It seems likely that the regulated variable would be some other correlate of body weight such as fat mass. Furthermore, the concept that calorie excess increases body temperature is controversial and complicated, and may depend on acute vs. chronic conditions. For example, rats maintained chronically on a high fat diet have reduced sympathetic outflow to BAT (Levin *et al *AJP, 1985 ^5^ ; Sakaguchi *et al* Physiol Behav, 1989 ^6^ ; Madden & Morrison AJP, 2016 ^7^ (my paper)).

**We have updated this section and included these references.**

1. Figure 2. The suggestion that the DMH contributes to energy expenditure and the PVH contributes to food intake is oversimplified. Clearly the paraventricular hypothalamus also influences energy expenditure (BAT) (see, Billington *et al*, 1994 ^8^ ; Madden & Morrison, 2009 ^9^ (my paper); Nakamura, 2017 ^10^ ).

**We have revised the figure to acknowledge that the distinction is relative rather than absolute.**

1. Page 8. “Selective reintroduction of melanocortin 4 receptors in the PVH normalizes the excess food intake but not the suppressed energy expenditure”. The selective reintroduction of MC4R in the cited work would be in the cell bodies in PVH not in afferent terminals to the PVH so the statement the MC4R is normalized in the PVH is an overinterpretation.

**We have revised this section.**

1. Page 10. Again the concept that the Arc to PVH projection is relevant to feeding and that the DMH projection modulates EE is overly simplistic. See the work of Shi *et al *J Neuroendo 2021 ^11^ ; Billington ^8^ ; Nakamura ^10^ .

**We have updated this section to acknowledge the complexity and that our description is a simplification.**
* *

1. Page 12, numbered point 2. Beta3 activation would not only activate WAT to suppress leptin but would also activate BAT to generate thermogenesis. 

**Yes, this is of course a good point we have included.**

1. The issue of the role of leptin in torpor is of high importance. Here again it calls into question the assertion that torpor in daily heterotherms and longer duration hibernation bouts are equivalent. Hibernation requires preparatory fattening which would result in elevated leptin levels, on the other hand daily torpor bouts appear to be blocked by leptin administration. 

**We agree and have revised our comments regarding hibernation and torpor to highlight this key difference.**

1. Page 14. Please provide a reference for the statements regarding the incidence of asystoles in torpor and following CHA.

**Included.**

1. The role of endogenous opioids in thermoregulation might benefit from a discussion of the role of kappa opioid receptors in high fat diet induced impairment of BAT activation. (see Conceicao *et al*, eNeuro, 2021 ^12^ (my paper)) and the parallels between animals maintained on a high fat diet and hibernation (see my paper, Madden, Temperature, 2017 ^13^ ).

**We have included reference to this paper. **

1. The suggestion that hypothermia-inducing DMH neurons are cholinergic is insightful and interesting. Nonetheless, the DMH neurons that express fos at torpor initiation are not cholinergic (Hitrec, 2019 ^14^ ).

**We have acknowledged this caveat.**

# Peer review: Reviewer response for version 1

**DOI:** 10.21956/wellcomeopenres.19215.r47147

**Published:** 2021-12-15

**Authors:** Sinisa Hrvatin, Senmiao Sun

**Affiliations:** 1 Whitehead Institute for Biomedical Research, Cambridge, MA, USA; 2 Massachusetts Institute of Technology, Cambridge, MA, USA; 3 Harvard Medical School, Boston, MA, USA

This article is an excellent review of the literature covering mechanisms that regulate the hypothermic and hypometabolic state of torpor. The review focuses on mouse torpor and neuronal mechanisms and makes relevant connections to literature on animal hibernation.

 Here are some specific comments/suggestions:

*Alongside hypothermia are commensurate reductions in metabolic, heart, and respiratory rates*

- The term “commensurate” suggests to me that the change in metabolic and heart rate is either proportional or caused by the change in temperature. Perhaps replace term.

*Torpor (or aestivation in the case of fish, reptiles, and amphibians) is a remarkably conserved behaviour seen in all classes of vertebrate life, including the three oldest branches of mammals: monotremes, marsupials, and placentals including primates, as illustrated in Figure 1:*

- We would not only briefly compare the biology of torpor with aestivation - the extent of the similarities and differences is not well understood. We suggest either not mentioning aestivation or elaborating much more on the differences/similarities.
- Figure 1 seems unrelated to this sentence.

*Figure 1*

- As you discuss later in the review, PACAP/BDNF neurons are predominantly glutamatergic (see Hrvatin, Sun *et al* 2020 ^1^, Takahashi *et al *2020 ^2^, Moffit 2018 ^3^).

*or simply increasing the energy costs of foraging (Schubert et al., 2010).*

- The observations in this story could be elaborated so they are not misunderstood by the reader. It appears that in this story mice lost weight during high-cost foraging which would have resulted in an energy deficient state that might be the reason animals entered torpor. 

*Hence, this behaviour may represent a fundamental physiological response that has been relatively recently switched off in those animals for which it is not an extant behaviour.*

- Cite references supporting this view and elaborate on term distant.

*torpor is engaged in any situation where the available food is insufficient for the metabolic demands of euthermia.*

- We would not agree with this statement as it implies that placing a mouse in a cage with no food would immediately engage torpor since this is a situation in which the food is non-existent (insufficient) to meet metabolic demands. Since hours of fasting or caloric deficit are required in WT mice,  it is not sufficient to expose an animal to a situation in which the food is insufficient.
- We also find it is difficult to generally state “any situation”.
- By many definitions, thermoneutrally-housed animals do not enter torpor.

*These gender differences might reflect the relatively lower energetic burden that normal activity and reproduction place on male compared to female mice, hence the need to conserve adipose tissue energy reserves may be greater for females*

- We believe “sex” is more appropriate than “gender”.
- Do male mice have more adipose tissue - could this be contributing as well?

*Although the trigger for torpor entry in mice usually originates from a fluctuating rather than predictable environmental stimulus, the timing of torpor entry is under circadian control.*

- We are sure which trigger the author is referring to. Lack of food in the environment might be predictable cue.

*with some evidence that torpor is engaged when food restriction or fasting decreases body weight to approximately 20g*

- There is literature on large and small mice entering torpor - 20g seems far from generalizable.

*Torpor typically persists for between four and six hours, often preceded by shallow, aborted or shorter bouts*

- If it is preceded by shorter bouts, it may be more accurate to say that it lasts minutes to hours.
- What is the difference between a short and an aborted bout?

*Hypothermia in a torpid animal could be achieved through three distinct mechanisms: increased thermal conductance to the environment; reduced target temperature; or reduced gain in the regulatory feedback system.*

- These concepts would need to be elaborated if the authors would like to discuss them. What is the meaning of target temperature (temperature setpoint)? What is the meaning of the gain of the feedback system? Which system? Although it is unclear whether it reflects the biology, We personally like the feedback framework with a temperature setpoint and gain. We don’t think however that this is an easily understood framework without further elaboration.

*The suppression of metabolism associated with torpor allows animals to tolerate profound reductions in cardiac output, respiratory rate, and, presumably, organ perfusion*

- Is it a suppression or could it simply be a decrease?

*This indicates a dominance of the parasympathetic nervous system during entry, followed by sympathetic nervous system activation during arousal from torpor, at least in terms of the heart.*

- For those less familiar with the functioning of the ANS elaborate on why this is the conclusion of the observation.

*More recently, the application of agonists or antagonists to cultured POA neurons that express calcium-sensitive fluorescent dyes established that central temperature-sensing mechanisms are mediated by the transient receptor potential M2 channel (TRPM2)*

- Mediated in part by this.

*Chemo- or optogenetic excitation of a warm-activated GABAergic projection from the ventral part of the lateral POA (vLPO) to the dorsomedial hypothalamic nucleus (DMH) suppresses thermogenesis and locomotion, while inhibiting the same projection induces the opposite effects.*

- Reference?

*Opto-activation of the warm-sensing GABAergic BDNF and PACAP-expressing cell bodies in the VMPO induces a drop in core body temperature, vasodilatation, and preference for a cooler environment.*

- See News and Views article by Saper, Machado ^4^ and single cell data/MERFISH by Moffit *et al *2018 ^3^ - these neurons are likely glutamatergic and co-express Gad1.

*One population responds to leptin, which is released from white adipose tissue (WAT) to signal satiety and replete WAT stores*

- Broadly the paragraph on Agrp/Pomc should make the point that ghrelin and leptin are but two signals that these cells sense/integrate.

*Ghrelin signals recent food intake and time since last meal. Sensory inputs signal the approaching likelihood of food.*

- Kindly add citation.

*These signals are generated by leptin, ghrelin, and sensory inputs, respectively.*

- There are also other humoral signals that these neurons sense.

*Given that leptin acts on POMC/α-MSH neurons in the arcuate, blocking this pathway should mimic a drop in leptin and therefore be pro-torpor. However Ay mice, which display ectopic AgRP production and through the antagonist effect of AgRP on melanocortin 4 receptors, impaired α-MSH – melanocortin signalling, in fact show a reduced tendency to torpor (Gluck et al., 2006).*

- Mention a caveat that compensation may play a role when examining these genetic models.

*However, while the core temperature of both leptin-treated and control male WT mice during 24 hours of fasting did decrease, it remained above 30°C, and therefore above commonly accepted thresholds for torpor.*

- The authors correctly note earlier in the paper that there is no standard definition for torpor. We would not consider 30°C a commonly accepted threshold for torpor.

*The arcuate is a key locus for NPY signalling, and selective ablation of ARC neurons with monosodium glutamate (MSG), supports a role for this nucleus in torpor.*

- We are not familiar with the earliest work on MSG and ARC neurons this but we’ve wondered since reading this original story how selective MSG actually is.
- RE torpor and sleep: Kindly add to discussion the recent story: “The relationship between fasting-induced torpor, sleep, and wakefulness in laboratory mice” ^5^.

*The data presented in the studies of Hrvatin, Takahashi, and Zhang represent significant advances in our understanding of torpor.*

- The Saper, Machado article “Flipping the switch on the thermoregulatory system" indicates that these neurons may also express EP3R (Machado J.Neurosci 2020) ^6^.

*We propose that there are two key features of torpor, which are relatively easily measured, and which might serve as a pragmatic proxy for identifying torpor: reduced body temperature with ongoing active thermoregulation such that a minimum (albeit adjusted) core temperature is defended; and controlled cardiovascular depression such that a characteristic heart rate versus body temperature hysteresis curve is observed*

- These two measures may not be sufficient and other measures like a change in movement/activity and posture may be relevant.

The discussion at the end can further highlight the fact that no stimulation of a neuronal population has recapitulated the full spectrum of natural torpor, including the animal posture. Therefore none of the identified neurons are likely to be the master switch controlling the entirety of the behavior and more work is needed to identify other neurons that regulate this process.

Is the review written in accessible language?

Yes

Are all factual statements correct and adequately supported by citations?

Partly

Are the conclusions drawn appropriate in the context of the current research literature?

Yes

Is the topic of the review discussed comprehensively in the context of the current literature?

Yes

Reviewer Expertise:

Torpor, hibernation, neuronal circuits regulating temperature and torpor

We confirm that we have read this submission and believe that we have an appropriate level of expertise to confirm that it is of an acceptable scientific standard.

## References

[1] : Neurons that regulate mouse torpor. *Nature* .583(7814) : 10.1038/s41586-020-2387-5 115-121 10.1038/s41586-020-2387-5 32528180PMC7449701

[2] : A discrete neuronal circuit induces a hibernation-like state in rodents. *Nature* .583(7814) : 10.1038/s41586-020-2163-6 109-114 10.1038/s41586-020-2163-6 32528181

[3] : Molecular, spatial, and functional single-cell profiling of the hypothalamic preoptic region. *Science* .2018;362(6416) : 10.1126/science.aau5324 10.1126/science.aau5324 PMC648211330385464

[4] : Flipping the switch on the body’s thermoregulatory system. *Nature* .2020;583(7814) : 10.1038/d41586-020-01600-5 34-35 10.1038/d41586-020-01600-5 32601488

[5] : The relationship between fasting-induced torpor, sleep, and wakefulness in laboratory mice. *Sleep* .2021;44(9) : 10.1093/sleep/zsab093 10.1093/sleep/zsab093 PMC843614433838033

[6] : EP3R-Expressing Glutamatergic Preoptic Neurons Mediate Inflammatory Fever. *The Journal of Neuroscience* .2020;40(12) : 10.1523/JNEUROSCI.2887-19.2020 2573-2588 10.1523/JNEUROSCI.2887-19.2020 PMC708353932079648

# Response

**Published:** 2021-12-15

**Authors:** Mike Ambler

**Affiliations:** School of Physiology, Pharmacology and Neuroscience, University of Bristol, UK

We thank the reviewer for their comments and suggestions. We broadly agree with what is said, and will update the manuscript to incorporate these points once further reviews are returned (in keeping with the guidance from the editors). 

 Regarding the specific point of the nature of the BDNF/PACAP projection from POA to DMH, we agree that recent evidence suggests a role for glutamatergic neurons particularly in torpor, but point out that earlier work from Tan *et al* (Cell, 2016) indicates that GABAergic neurons play a role in the context of thermoregulation. We will update the manuscript to include the suggestion that there is evidence for both.

# Response

**Published:** 2022-03-09

**Authors:** Mike Ambler

**Affiliations:** School of Physiology, Pharmacology and Neuroscience, University of Bristol, UK

This article is an excellent review of the literature covering mechanisms that regulate the hypothermic and hypometabolic state of torpor. The review focuses on mouse torpor and neuronal mechanisms and makes relevant connections to literature on animal hibernation.

 Here are some specific comments/suggestions:

 Alongside hypothermia are commensurate reductions in metabolic, heart, and respiratory rates

- The term “commensurate” suggests to me that the change in metabolic and heart rate is either proportional or caused by the change in temperature. Perhaps replace term

**We have removed the term to “commensurate”.**

 Torpor (or aestivation in the case of fish, reptiles, and amphibians) is a remarkably conserved behaviour seen in all classes of vertebrate life, including the three oldest branches of mammals: monotremes, marsupials, and placentals including primates, as illustrated in Figure 1:

- We would not only briefly compare the biology of torpor with aestivation - the extent of the similarities and differences is not well understood. We suggest either not mentioning aestivation or elaborating much more on the differences/similarities.

**In the interest of keeping the manuscript to a manageable length we have removed reference to aestivation.**

- Figure 1 seems unrelated to this sentence.

**We have removed this error.**

 Figure 1

- As you discuss later in the review, PACAP/BDNF neurons are predominantly glutamatergic (see Hrvatin, Sun et al 2020 ^1^, Takahashi et al 2020 ^2^, Moffit 2018 ^3^ ).

**We have adjusted the discussion in this section to acknowledge that the use of GAD to identify GABAergic cells might misidentify neurons as inhibitory and have referred to the article from Saper & Machado.**

 or simply increasing the energy costs of foraging (Schubert et al., 2010).

- The observations in this story could be elaborated so they are not misunderstood by the reader. It appears that in this story mice lost weight during high-cost foraging which would have resulted in an energy deficient state that might be the reason animals entered torpor. 

**We have adjusted the text to clarify.**

 Hence, this behaviour may represent a fundamental physiological response that has been relatively recently switched off in those animals for which it is not an extant behaviour.

- Cite references supporting this view and elaborate on term distant.

**We have adjusted this paragraph to include the references at the correct position.**

 torpor is engaged in any situation where the available food is insufficient for the metabolic demands of euthermia.

- We would not agree with this statement as it implies that placing a mouse in a cage with no food would immediately engage torpor since this is a situation in which the food is non-existent (insufficient) to meet metabolic demands. Since hours of fasting or caloric deficit are required in WT mice, it is not sufficient to expose an animal to a situation in which the food is insufficient.
- We also find it is difficult to generally state “any situation”.
- By many definitions, thermoneutrally-housed animals do not enter torpor.

**Have removed this statement.**

 These gender differences might reflect the relatively lower energetic burden that normal activity and reproduction place on male compared to female mice, hence the need to conserve adipose tissue energy reserves may be greater for females

- We believe “sex” is more appropriate than “gender”.
- Do male mice have more adipose tissue - could this be contributing as well?

**Have replaced gender with sex.**

 Although the trigger for torpor entry in mice usually originates from a fluctuating rather than predictable environmental stimulus, the timing of torpor entry is under circadian control.

- We are sure which trigger the author is referring to. Lack of food in the environment might be predictable cue.

**We have rephrased this sentence to indicate that lack of food might be an unpredictable cue (while acknowledging that it could also be predictable).**

 with some evidence that torpor is engaged when food restriction or fasting decreases body weight to approximately 20g

- There is literature on large and small mice entering torpor - 20g seems far from generalizable.

**We have highlighted the fact this may not be generalizable.**

 Torpor typically persists for between four and six hours, often preceded by shallow, aborted or shorter bouts

- If it is preceded by shorter bouts, it may be more accurate to say that it lasts minutes to hours.
- What is the difference between a short and an aborted bout?

**We have removed the word aborted.**

 Hypothermia in a torpid animal could be achieved through three distinct mechanisms: increased thermal conductance to the environment; reduced target temperature; or reduced gain in the regulatory feedback system.

- These concepts would need to be elaborated if the authors would like to discuss them. What is the meaning of target temperature (temperature setpoint)? What is the meaning of the gain of the feedback system? Which system? Although it is unclear whether it reflects the biology, We personally like the feedback framework with a temperature setpoint and gain. We don’t think however that this is an easily understood framework without further elaboration.

**We have expanded somewhat but in the interest of keeping the manuscript at a manageable length we have not included a detailed discussion, believing that the interested reader will follow the citations.**

 The suppression of metabolism associated with torpor allows animals to tolerate profound reductions in cardiac output, respiratory rate, and, presumably, organ perfusion

- Is it a suppression or could it simply be a decrease?

**We believe metabolism is actively suppressed beyond the reduction that occurs purely due to Q10 effects – although we acknowledge that the evidence for this comes largely from hibernating bears and not from daily torpor.**

 This indicates a dominance of the parasympathetic nervous system during entry, followed by sympathetic nervous system activation during arousal from torpor, at least in terms of the heart.

- For those less familiar with the functioning of the ANS elaborate on why this is the conclusion of the observation.

**We believe the interested reader can follow the citations for a detailed discussion of this topic.**

 More recently, the application of agonists or antagonists to cultured POA neurons that express calcium-sensitive fluorescent dyes established that central temperature-sensing mechanisms are mediated by the transient receptor potential M2 channel (TRPM2)

- Mediated in part by this.

**We have revised this sentence.**

 Chemo- or optogenetic excitation of a warm-activated GABAergic projection from the ventral part of the lateral POA (vLPO) to the dorsomedial hypothalamic nucleus (DMH) suppresses thermogenesis and locomotion, while inhibiting the same projection induces the opposite effects.

- Reference?

**Zhao 2017**

 Opto-activation of the warm-sensing GABAergic BDNF and PACAP-expressing cell bodies in the VMPO induces a drop in core body temperature, vasodilatation, and preference for a cooler environment.

- See News and Views article by Saper, Machado ^4^ and single cell data/MERFISH by Moffit et al 2018 ^3^ - these neurons are likely glutamatergic and co-express Gad1

**Have updated this section to include the nuances of the possibility that GAD is not a useful marker of GABAergic neurons in isolation, and hence thee neurons may in fact be glutamatergic.**

 One population responds to leptin, which is released from white adipose tissue (WAT) to signal satiety and replete WAT stores

- Broadly the paragraph on Agrp/Pomc should make the point that ghrelin and leptin are but two signals that these cells sense/integrate.

Ghrelin signals recent food intake and time since last meal. Sensory inputs signal the approaching likelihood of food.

- Kindly add citation.

**We have added a citation, although this paragraph really represents a synthesis of the preceding paragraphs.**

 These signals are generated by leptin, ghrelin, and sensory inputs, respectively.

- There are also other humoral signals that these neurons sense.

**We have acknowledged that this is just a part of the story.**

 Given that leptin acts on POMC/α-MSH neurons in the arcuate, blocking this pathway should mimic a drop in leptin and therefore be pro-torpor. However Ay mice, which display ectopic AgRP production and through the antagonist effect of AgRP on melanocortin 4 receptors, impaired α-MSH – melanocortin signalling, in fact show a reduced tendency to torpor (Gluck et al., 2006).

- Mention a caveat that compensation may play a role when examining these genetic models.

**Have included.**

 However, while the core temperature of both leptin-treated and control male WT mice during 24 hours of fasting did decrease, it remained above 30°C, and therefore above commonly accepted thresholds for torpor.

- The authors correctly note earlier in the paper that there is no standard definition for torpor. We would not consider 30°C a commonly accepted threshold for torpor.

**We have removed this statement.**

 The arcuate is a key locus for NPY signalling, and selective ablation of ARC neurons with monosodium glutamate (MSG), supports a role for this nucleus in torpor.

- We are not familiar with the earliest work on MSG and ARC neurons this but we’ve wondered since reading this original story how selective MSG actually is.
- RE torpor and sleep: Kindly add to discussion the recent story: “The relationship between fasting-induced torpor, sleep, and wakefulness in laboratory mice” ^5^.

**Added.**

 The data presented in the studies of Hrvatin, Takahashi, and Zhang represent significant advances in our understanding of torpor.

- The Saper, Machado article “Flipping the switch on the thermoregulatory system" indicates that these neurons may also express EP3R (Machado J.Neurosci 2020) ^6^.

We propose that there are two key features of torpor, which are relatively easily measured, and which might serve as a pragmatic proxy for identifying torpor: reduced body temperature with ongoing active thermoregulation such that a minimum (albeit adjusted) core temperature is defended; and controlled cardiovascular depression such that a characteristic heart rate versus body temperature hysteresis curve is observed

- These two measures may not be sufficient and other measures like a change in movement/activity and posture may be relevant.

**Agreed, however we propose that a pragmatic approach might focus on these characteristics.**

 The discussion at the end can further highlight the fact that no stimulation of a neuronal population has recapitulated the full spectrum of natural torpor, including the animal posture. Therefore none of the identified neurons are likely to be the master switch controlling the entirety of the behavior and more work is needed to identify other neurons that regulate this process.

**We have highlighted this point.**
